# Genome-wide methylated CpG island profiles of melanoma cells reveal a melanoma coregulation network

**DOI:** 10.1038/srep02962

**Published:** 2013-10-16

**Authors:** Jian-Liang Li, Joseph Mazar, Cuncong Zhong, Geoffrey J. Faulkner, Subramaniam S. Govindarajan, Zhan Zhang, Marcel E. Dinger, Gavin Meredith, Christopher Adams, Shaojie Zhang, John S. Mattick, Animesh Ray, Ranjan J. Perera

**Affiliations:** 1 Sanford-Burnham Medical Research Institute, Orlando FL 32827 USA; 2 Department of Electrical Engineering and Computer Science, University of Central Florida, Orlando FL 32816 USA; 3 Cancer Biology Program, Mater Medical Research Institute, South Brisbane, Queensland 4101, Australia; 4 School of Biomedical Sciences, University of Queensland, Brisbane, Queensland 4072, Australia; 5 Garvan Institute of Medical Research, Darlinghurst NSW 2010, Australia; 6 Life Technologies, Carlsbad CA 92008 USA; 7 School of Applied Life Sciences, Keck Graduate Institute, Claremont CA 91711 USA; 8 These authors contributed equally to this work.

## Abstract

Metastatic melanoma is a malignant cancer with generally poor prognosis, with no targeted chemotherapy. To identify epigenetic changes related to melanoma, we have determined genome-wide methylated CpG island distributions by next-generation sequencing. Melanoma chromosomes tend to be differentially methylated over short CpG island tracts. CpG islands in the upstream regulatory regions of many coding and noncoding RNA genes, including, for example, *TERC*, which encodes the telomerase RNA, exhibit extensive hypermethylation, whereas several repeated elements, such as LINE 2, and several LTR elements, are hypomethylated in advanced stage melanoma cell lines. By using CpG island demethylation profiles, and by integrating these data with RNA-seq data obtained from melanoma cells, we have identified a co-expression network of differentially methylated genes with significance for cancer related functions. Focused assays of melanoma patient tissue samples for CpG island methylation near the noncoding RNA gene *SNORD-10* demonstrated high specificity.

Melanoma, a lethal form of skin cancer whose risk is increasing in the United States, is difficult to diagnose early and has been refractory to targeted chemotherapy^1^. While it is generally true that melanomas are caused by ultraviolet B induced lesions in melanocytes of the skin, the exact molecular events leading to melanoma production are yet unknown^2^. Over 90% of melanomas are independent of TP53 gene mutation^3^, suggesting pathways other than those involved in p53 mediated DNA repair or abrogation of apoptosis by p53 are generally not directly involved. Early events in melanoma induction involve epithelial-mesenchymal transition, and both protein coding and noncoding RNA genes are important^4^. Several protein-coding genes have been identified as candidate drug targets and potential early biomarkers for aggressive melanoma^5^,^6^,^7^,^8^,^9^,^10^,^11^, of which several exhibit distinct expression signatures among a variety of malignant metastatic melanomas and their benign forms^12^,^13^,^14^. However, there are unquestioned needs for further understanding the molecular mechanisms of melanoma development and progression, and to develop additional sensitive and specific early diagnostic biomarkers. An important question for both understanding the biology of melanomas and for better biomarker discovery is the role of epigenetic modification, such as CpG methylation and chromatin protein modification, in differential expression of genes during melanoma initiation and progression.

Aberrant methylation of promoter CpG islands resulting directly from the activities of cytosine DNA methyltransferases^15^ is frequently involved in cancers^16^. Three DNA methyltransferase genes, DNMT1, DNMT3A, and DNMT3B, are directly responsible for DNA CpG island methylation^17^,^18^, and approximately 50 genes are regulated, at least in part, by hypermethylation of CpG islands in their respective regulatory regions in human melanomas^19^. Of these, RASSF1 is a hallmark gene for abnormal methylation in many cancers including metastatic melanomas^20^,^21^. Among genes that exhibit differential CpG island modification in their putative regulatory regions are the non-protein coding RNAs (ncRNAs). Small ncRNA molecules such as microRNAs (miRNAs) have garnered increasing attention for their potential roles in tumorigenesis^22^,^23^,^24^,^25^,^26^, including in melanoma^11^,^27^,^28^,^29^,^30^,^31^,^32^,^33^,^34^. miRNAs influence cancer development by regulating transcription and translation of both tumor suppressor genes and oncogenes^34^,^35^,^36^,^37^,^38^,^39^,^40^. Since miRNA precursor genes are usually nested within other protein coding genes, often within intron sequences, the misregulation of these protein-coding genes by epigenetic mechanisms is expected to cause aberrant regulation of the miRNA target genes. miRNA gene silencing by CpG island methylation has been reported in several cancers^41^,^42^,^43^, although little is known in this regard for melanomas^44^. Our previous results with melanoma cell lines and clinical samples demonstrated that the expression of several miRNA species is epigenetically regulated in human melanoma and we have recently reported on the molecular mechanisms by which two such miRNAs, miR-375 and miR-34b, might affect melanoma development in humans^45^,^46^.

While it is generally thought that differentiated melanocytes must first dedifferentiate and then undergo transformation due to successive mutations, there are also reports of melanoma stem cells^47^,^48^. Recently, a mutational signature of melanoma cells sampled from multiple melanoma subtypes has been described^49^,^50^,^51^. Additionally, a recent landmark study^50^ finds numerous mutations distributed over the melanoma genome, including many inter and intra-chromosomal rearrangements reminiscent of chromothripsis^52^ and numerous single base changes that bear the preferential C → T transition bias expected of UV mutagenesis (including in BRAF, PTEN, and PTEN-interacting genes). These studies underscore the significant role that genome rearrangements and point mutations, presumably induced by UV radiation, evidently play in melanoma development. It not clear, however, how these mutations contribute to melanoma development, and whether (or what fraction of) UV induced mutations are the primary causes or indirect effects of epigenetic reprogramming that evidently accompanies melanoma progression. Here we examine a related question: do melanocytes and melanoma cells that have been established from distinct melanoma stages differ in their epigenetic signatures? By understanding the epigenetic changes in the coding and noncoding RNA genes during melanoma progression it might be possible to obtain an additional perspective of the molecular mechanisms and point towards more effective biomarker development for melanoma prognosis. The melanoma epigenome is complex^53^; here we focus on a small subset of the melanoma epigenome—the genome-wide CpG island methylation status.

Characterization of methylated CpG islands genome-wide has been conducted in the past by either direct bisulfite sequencing^54^, by methylation sensitive restriction endonuclease site mapping^55^, high resolution melt sequencing^56^,^57^, and by enriching methylated cytidines using a monoclonal antibody^58^,^59^. Methyl binding domain 2 protein (MBD2) has been used to specifically pull-down methylated CpG islands with high efficiency and specificity in a focused study of CpG island methylation of selected genes^60^.

Compared to other methods, such as direct bisulfite sequencing, MBD2 allows rapid and selective identification of highly methylated CpG islands^61^,^62^, but suffers from under-estimation of the total spectrum of CpG methylation because CpG methylation in non-island sites are generally ignored. However, the relative merits of these distinct techniques for genome wide methylome analysis are currently difficult to evaluate because a head-to-head comparison of these assays has not yet been performed^63^. We have used MBD2 pull down for its high throughput capabilities and also because of its recent reported use in characterizing the methylomes of cancer cell lines other than melanoma^62^. Here, we report for the first time the characterization of the global melanoma CpG island methylome by MBD2-mediated pull-down, followed by next generation sequencing performed on samples from four different melanoma cell lines. We have characterized the CpG island methylation patterns in the regulatory regions of coding and noncoding RNA genes in melanoma-derived cell lines, melanoma patient samples, melanocytic nevi and normal melanocytes. The large number of coding and noncoding methylated regions identified in this study will be a resource for cancer biologists interested in the epigenomics of melanoma progression. We have discovered that under-methylated CpG islands are associated with certain Alu elements and retrotransposable elements in late stage melanoma cell lines in contrast with those in early stage melanoma cells and melanocytes. Finally, we present genome wide methyl CpG island signatures that are characteristic of melanocytes, of early stage and of late stage melanomas, respectively. These results are in agreement with the notion that melanocytes undergo progressive hypomethylation across large tracts of CpG islands genome-wide as these are transformed into early stage melanomas, followed by extensive hypermethylation as late stage melanomas develop. Coupled with the recent report of pervasive transposable element movements in several epithelial cancer genomes and their correlation with regions of cancer-specific DNA hypomethylation tracts^64^, and the presumed role of these elements in causing mutagenesis, our results underscore the importance of methyl CpG dynamics during melanoma progression.

## Results

### Genomic CpG islands are differentially methylated in melanoma cells

**Figure 1:**
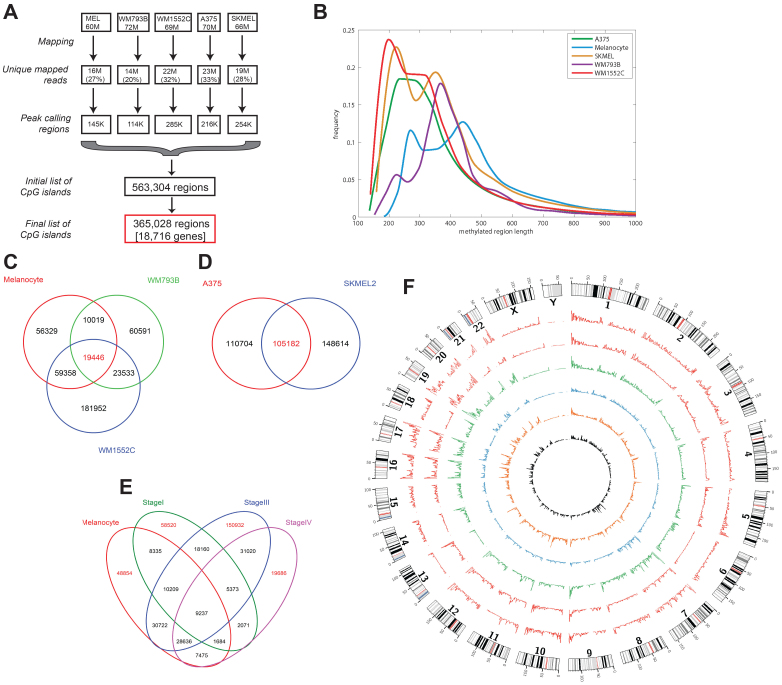
Characterization of genome-wide methylation patterns in melanoma cell lines. (A) The overall workflow, and results of next-generation sequencing and analysis of MBD2-pulldown DNA. (B) Frequency distributions of sizes of the MBD2-pulldown DNA across five cell lines. The methylated regions were defined by MACS peak calling of the MBD2-pulldown DNA against input (non-enriched) DNA. (C) & (D) Venn diagram of the number of MBD2-pulldown regions shared or unique among the various cell lines. (E) Venn diagram describing the number of MBD2-pulldown DNA regions partitioned among various cell lines, which defines a unique set of stage IV specific MBD2-pulldown DNA regions. (F) Genome-wide distribution of differential methylated regions (as defined as the MBD2-pulldown DNA). The outermost circle displays the human chromosomes. The inner circles represent the genome-wide distribution of MBD2-pulldown DNA. The height of the histogram bins indicates the density of methylated regions. From the inner most circle, which represents the distribution of MBD2-pulldown DNA in 5AzadC treated WM1552C (black), the circles from inside going outside represent HEM-1 (orange), WM793B (blue), WM1552C (green), A375 (red) and SKEML2 (red).

To identify the highly methylated regions in the melanoma genome, we used methyl-CpG-binding protein (MBD2) to pull down methylated CpG islands of human melanoma cell lines and normal melanocytes (HEM-l), and the enriched sequences were subjected to deep-sequencing (see Methods). The melanoma cell lines were obtained from (A) stage I primary melanoma vertical growth phase: WM793B; (B) invasive metastatic melanoma stage III (radial growth phase): WM1552C; and (C) distant metastatic stage IV: A375 and SK-MEL2. The raw DNA sequence data were first examined for their sequence quality and then the reads with average quality values over 20 (see Methods) were mapped to the version hg19 reference human genome. MBD2 enriched regions were further analyzed for cell-type specific methylation patterns. The total numbers of initial peak sequence calls (563 K) (Fig. 1A) were further partitioned and mapped to regions 3 kb upstream of transcriptional start site (TSS), and 3 kb downstream of transcription end sites (TES) of protein coding genes. In total, 365 K peak MBD2-enriched sequenced regions could be aligned to 18,716 genes based on the above criteria (Fig. 1A). The remaining robust MBD2-pulldown sequence peaks either mapped to unannotated regions, or mapped to ncRNA and/or repetitive DNA regions (see later). Figure 1B shows the frequency distribution of unique MBD2-pulldown DNA of various cell lines. Melanocytes (control cells) showed relatively low levels of MBD2-pulldown DNA compared to those in the melanoma cells, however, there were two distinct peaks at 275 and 450 bp, respectively. By contrast, the stage III line WM1552C and stage IV lines SK-MEL2 and A375 displayed enriched MBD2-pulldown tracts of shorter lengths on average than those seen with the stage I line WM793B. Melanocytes (145,221 peaks) and the stage I melanoma line WM793B (113,657 peaks) showed half as many MBD2-pulldown peaks than the other melanoma cell lines (WM1552C, 284,533; SK-MEL2, 263,796; and A375, 216,021 peaks, respectively. Supplementary Table S1). Furthermore, the cell lines that were enriched for the total segments of MBD2-pulldown DNA had on average shorter DNA stretches than those with fewer segments of MBD2-pulldown DNA. For most cell lines two peaks, one at ~ 200–300 bp and another at ~ 350–450 bp, or a broad peak with a hint of a shoulder (for A375) could be discerned.

**Figure 2:**
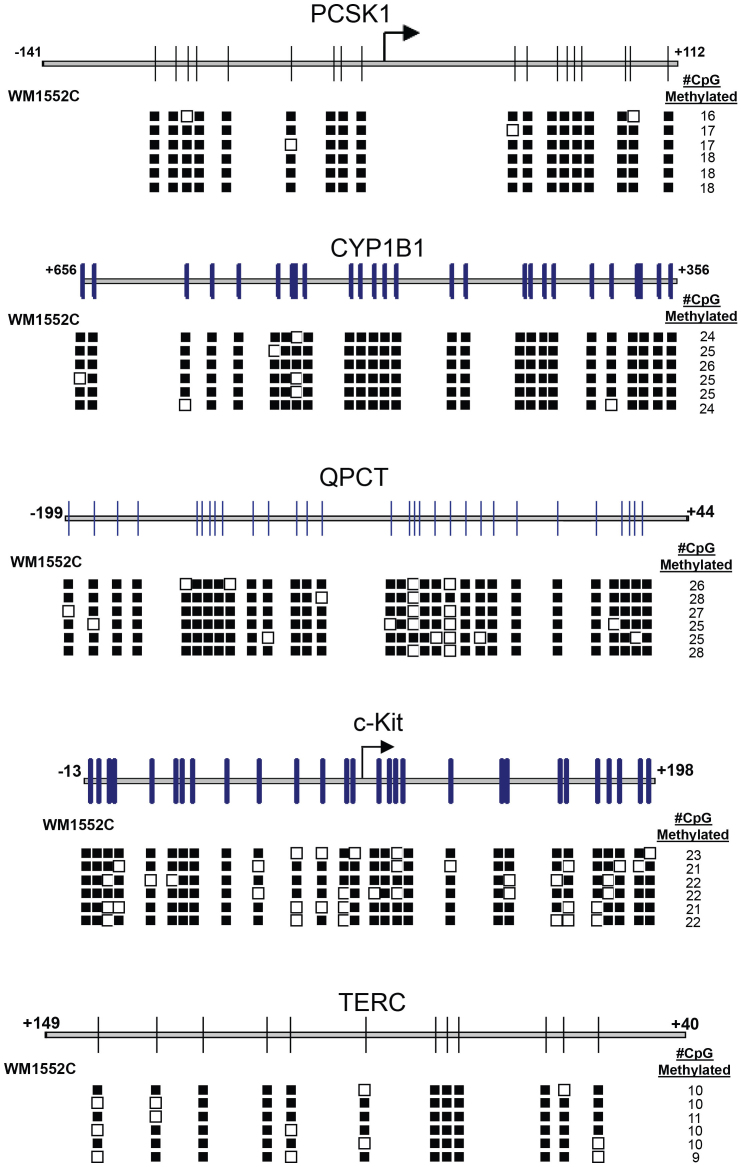
DNA fragments pulled-down with MBD2 are enriched for methylated CpG islands. Results of bisulfite sequencing of MBD2-pulldown DNA near five genes are shown. Methylated CpG sequences are shown as closed boxes (unmethylated sequences are open boxes). The number at the right of each row corresponds to the number of CpG in the region that was methylated, with the total number of CpG represented as the total number of boxes in each row. Each row is an independent experiment.

To determine whether the MBD2-enriched DNA corresponded to methylated CpG islands, we conducted targeted bisulphite sequencing of MBD2-pulldown DNA at five genes (PCSK1, CYP1B1, QPCT, c-KIT, and TERC). These genes were selected on the basis of methyl-CpG levels in their upstream regulatory regions shown in the MBD2-pulldown experiments and of their existence in previous reports of differential methylation in melanoma or cancer cells^53^,^65^. Here we found a complete correlation between hypermethylated CpG islands in the MBD2-pulldown DNA and high level of CpG methylation by bisulfite sequencing (Fig. 2). These results show that for all practical purposes, the MBD2-pulldown DNA corresponds to highly methylated CpG island DNA.

Figure 1C shows the number of highly methylated CpG islands shared or unique among the melanocyte, WM793B (stage I) and WM1552C (stage III) cell lines. Likewise in Figure 1D, the numbers of methyl-CpG islands in 2 stage IV lines are compared, which shows that 105,182 methylated CpG islands are in common. To identify the methyl CpG islands unique to stage IV lines, we intersected this core set of 105,182 islands with those of melanocytes, stage I, and stage III lines, and obtained a set of 19,686 islands (Fig. 1E). Of these, 14,849 regions could be aligned to 5,700 annotated genes with the condition that the query regions were located within a sequence window containing 3 kb upstream of TSS and 3 kb downstream of TES, including the entire set of introns and exons for every gene. When the sequence windows were reduced to regulatory region (2 kb upstream and 1 kb downstream of TSS), 885 regions could be aligned to 821 unique genes. Functional analysis revealed that these 821 genes are statistically enriched for important developmental pathways and cell biological functions, including stem cell differentiation (p = 3.09 × 10^−4^), cellular movement (p = 9.59 × 10^−5^), cell morphology (p = 1.34 × 10^−3^), connective tissue development (p = 1.34 × 10^−3^) and embryonic development (p = 1.34 × 10^−3^) (Supplementary Fig. S1A–D). Figure 1F shows circular plots of chromosome-wide methyl CpG island peaks for all cell lines. Some chromosomes, such as chromosomes 4, 6, 8 and 13, appear to have on average fewer methyl CpG island peaks compared to several others, such as chromosomes 1, 16, 17, 19, and 20.

### Methyl CpG islands in genic and non-genic regions

**Figure 3:**
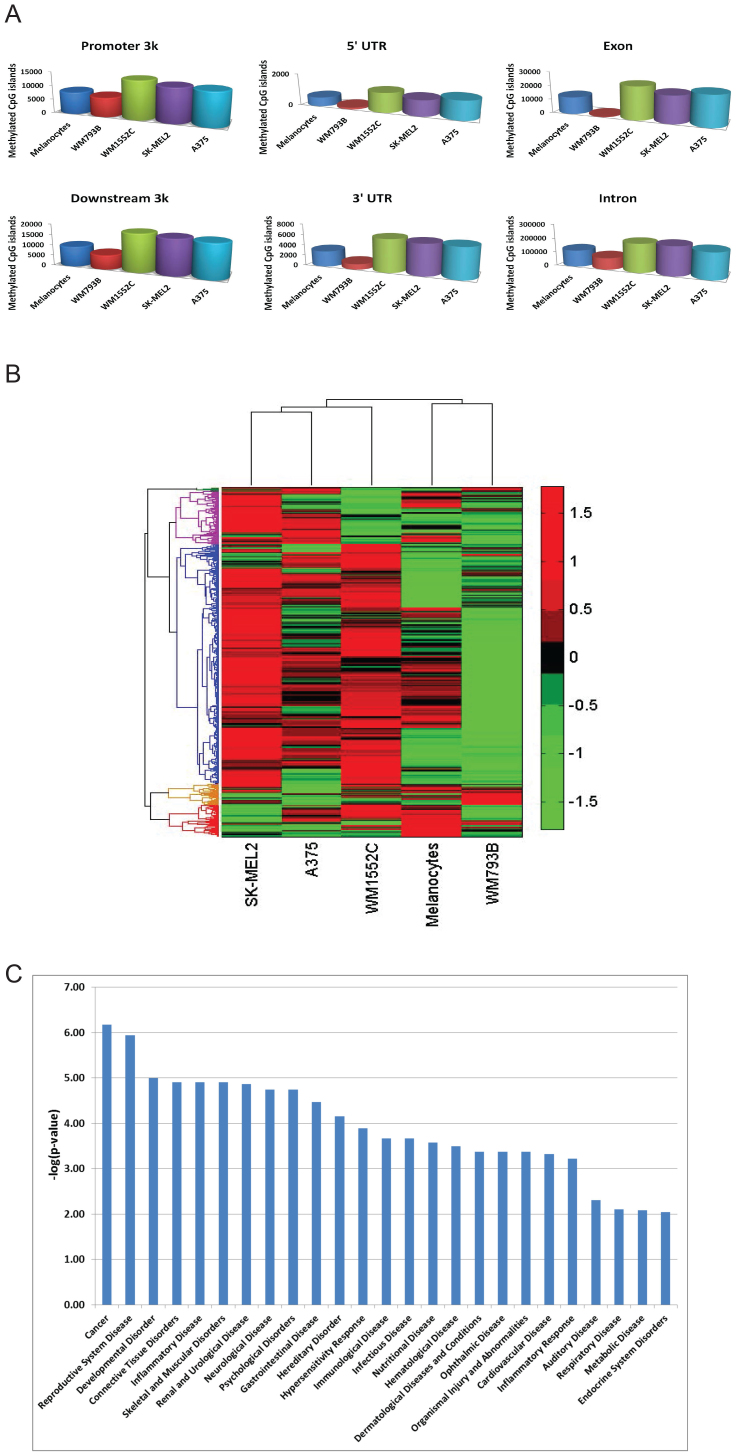
Functional significance of methylated CpG islands in melanoma cells. (A) The functional class distribution of CpG methylation in the five cell lines. Gene annotation was taken from Refseq at UCSC genome browser. Promoter region was defined as 3000 bp upstream of the transcription starting site (TSS), and the downstream region is defined as 3000 bp downstream of translation stop sequence. (B) Heatmap of methylation fold change in ± 1000 bp windows centered at TSS for cancer related genes in the five cell lines. The rows represent 3,688 cancer-related genes downloaded from the CancerGene database by Higgin et al.^66^, which were filtered for significant methyl CpG levels in melanoma lines relative to HEM-1. Color scale on the right represents MBD2-enrichment, i.e., methyl CpG, fold change. (C) Functional enrichment analysis of diseases and disorders of the gene set in (B).

We sought to ascribe the CpG islands to genic and non-genic regions in the following way. The genomic regions were separated into six functional categories: promoter region (up to 3 Kb upstream of transcription start sites or TSS), 5'UTR, exon, intron, 3'UTR, and downstream region (up to 3 Kb downstream of the stop codon). Some of the downstream sequence regions overlapped with 3'UTR of a given gene. For peaks that belonged to multiple genomic regions, the length of the overlapped regions broke the ties. For example, if a peak overlapped 300 bp with a gene's promoter region and 400 bp with another gene's intronic region, the peak was assigned as an intronic region. Each peak was assigned to a specific genomic interval and the highest numbers of tag counts were seen between 100 bp and 400 bp tag lengths, but the lengths of the methyl-CpG islands extended 100–800 bp (Fig. 1B). Tag counts of all melanoma cell lines and melanocytes were normalized to that of WM1552C input (non-enriched) DNA. Figure 3A summarizes these results. In general, the extent of methyl CpG islands is higher in the stage III and stage IV cell lines than that of any other cell lines. The exon and intron regions of the stage I cells have significantly reduced methylated CpG islands compared to the corresponding regions of any other cell line including the precursor melanocytes (Supplementary Table S2). With respect to the distribution of methyl CpG islands, the stage III and IV cell lines were mutually indistinguishable, but these two stages were distinguishable from the melanocytes and the stage I cell line in all of the genomic intervals monitored. That these differences are biologically significant is shown by clustering the methyl CpG-enriched DNA from ± 1000 bp of the TSS of cancer-related genes extracted from the datasets^66^ (Fig. 3B): the extent of methylated CpG islands corresponding to the promoter regions of cancer-related genes in the two stage IV lines clustered together, followed by those in the stage III line; those in the stage I line were the furthest away from the two stage IV lines. These results demonstrate that in as much as known cancer-related genes are concerned, their regulatory regions are differentially methylated at the CpG islands in stage III and IV cells relative to melanocytes and the stage I cell line.

### Reversal of methylation at CpG islands by DNA methyl transferase inhibition reveals a transcriptionally co-regulated gene network

In this section we address the functional significance of differential promoter hypermethylation in stage III–IV cells. We first depleted methyl-CpG of the stage III WM1552C cells with 5AzadC treatment and compared the relative abundance of methyl-CpGs that could be depleted in these cells with those in untreated cells. 5AzadC concentration was chosen on the basis of our previous studies, which showed a nearly complete loss of CpG methylation combined with induction of gene expression in CpG-dense promoters^45^,^46^. We pulled down MBD2 bound DNA, and subjected these to next generation sequencing as described before. In the total sequenced dataset, we focused specifically on methyl-CpG stretches located within 2 kb upstream and 1 kb downstream of the transcription start site (TSS) of coding genes. This process generated 7,590 regions corresponding to 5,637 genes. We further filtered this number by incorporating nearby CpG island information from the UCSC human genome database, and promoter information from the eukaryotic promoter database^67^, in a 1 kb upstream region of each TSS. This yielded 583 highly methylated upstream regulatory regions corresponding to 581 genes. Figure 3C illustrates the enrichment of Gene Ontology (GO) functional category of Diseases and Disorders. Enrichment data for other functional categories (physiological system development and molecular and cellular function) are provided in Supplementary Figure S2 and a list of these 583 regions (581 genes) is in Supplementary Table S3.

**Figure 4:**
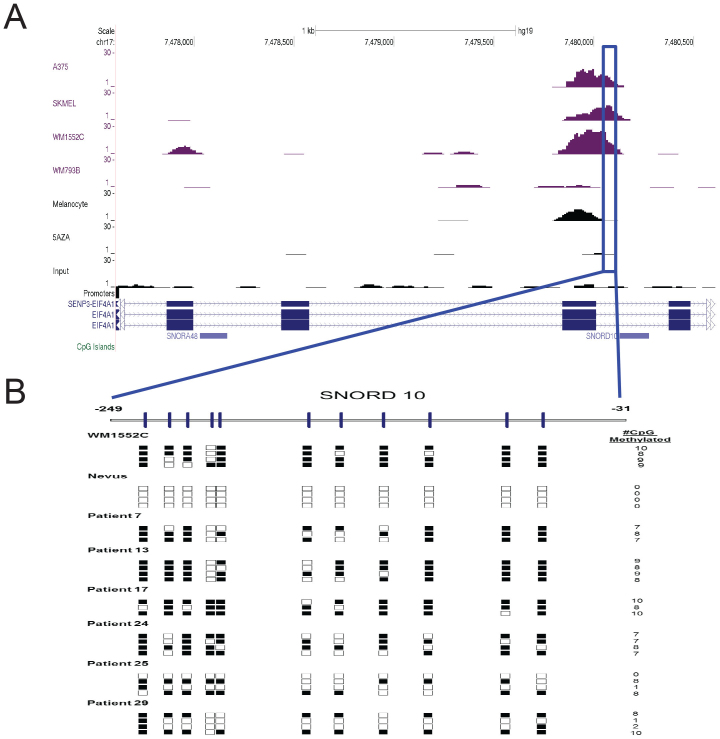
CpG methylation status near *SNOR-10* gene in different patient samples. (A) depicts the results obtained with next-generation sequencing of MBD2-enriched DNA in the five cell lines, plus those in WM1552C cells treated with 5AzadC and those in the input DNA. (B) A region between −249 bp and −31 bp of the TSS of SNORD-10 is shown magnified. Methylation status of this region in the DNA obtained from various metastatic melanoma patient samples or in non-melanoma nevi was determined by bisulfite sequencing (see Methods). Each box is a CpG; open boxes are unmethylated, filled boxes are methyl CpG sequences. Numbers at the right represent the number of methyl CpG detected in the region corresponding to each row (independent sequence analysis).

To validate whether the methylated CpG islands that could be erased by treatment with 5AzadC correlated with silenced genes in melanoma cells, we determined the extent of CpG island methylation in the regulatory regions of the set of 581 genes with RNA abundance data (RNA-seq) from melanocytes and WM1552C (Supplementary Table S3). Here also, the two stage IV cell lines (A375, and SK-MEL2) resembled the CpG island methylation patterns of the stage III line WM1552C. Interestingly, the CpG islands in the upstream region of PCSK1 are methylated in WM1552C but not in any of the other tested cell lines (Supplementary Fig. S3A). We also examined the methylation status of two noncoding RNA genes—the telomerase RNA gene, TERC and the snoRNA gene SNORD-10. The reason for choosing these two genes was that the expression of these transcripts showed a considerable down-regulation in WM1552C and transcript levels of both genes were up-regulated upon treatment with 5AzadC. The upstream putative promoter regions of both TERC and SNORD-10 ncRNA are highly methylated at the CpG islands, and are reversed upon treatment with 5AzadC (Supplementary Fig. S3B and Fig. 4A).

We queried a database of cancer-related human gene expression^68^ (containing 1,180,670 interactions) for correlated gene expression levels between all pairs of genes from the list of 581 genes above. This yielded a fully connected network of 554 co-expressed genes (Fig. S4) (and 68 unconnected genes), suggesting that this subset of 554 genes is under coordinate regulation by a common set of transcription factors or epigenetic regulators. This co-expression network fits a power law degree distribution (exponent γ = −1.6; R^2^ = 0.803), suggesting that it is a biologically robust network^69^. The 554-member co-expression network was significantly enriched (Benjamini-Hochberg (B-H) corrected *P* = 1.47 × 10^−8^) for the Gene Ontology (GO) molecular functional annotation of “sequence-specific DNA binding” (GO:0043565) (36/581 input query genes), and “sequence-specific DNA-binding transcriptional factor” (GO:0003700) (B-H corrected *P* = 1.16 × 10^−6^; 48/581 query genes) as anticipated for a transcriptional co-expression network. Moreover, the network was also enriched (B-H corrected *P* = 3.4 × 10^−8^) for “alpha-2 macroglobulin receptor-associated protein activity” (GO:0005515) and contains 191/581 input genes in this annotation cluster. The full set of GO enrichment categories along with B-H corrected P values are provided in Supplementary Table S4.

**Figure 5:**
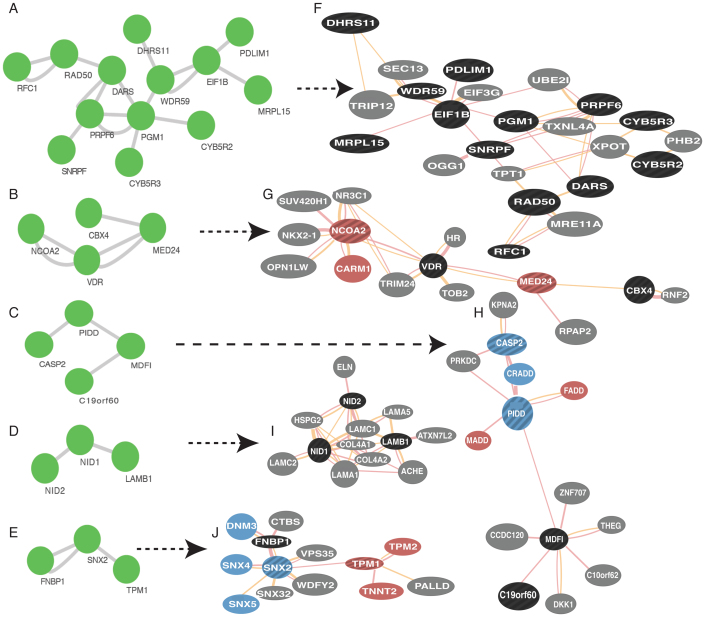
MBD2-enriched DNA from WM1552C cells defines a large co-expression network with cancer and metastasis-related genes. A co-expression network formed by the genes that exhibit (a) enrichment for MBD2-pulldown relative to HEM-l, (b) differential RNA expression levels measured by RNA-seq relative to HEM-l, and (c) included in cancer-related genes by Higgins et al^66^. (A)–(E) Proteins of the connected component in the co-repression network (Fig. S4) that are known to interact as members of known protein complexes. (F)–(J) Protein complexes constructed by extending the core protein interaction networks in (A–E) as seeds by incorporating known first-degree interaction neighbors of the member proteins. The protein modules are enriched for RNA-dependent DNA replication (F), ligand-dependent transcription factor activity (G), DNA damage response and cell death (H), basal lamina/substrate-dependent cell movement (I), and actin filament sliding/cell movement (J) (see text for *P*-values).

To further investigate the possible functions of this group of genes, we queried the human protein-protein interaction database with this gene set for inclusion of the member gene products in known protein complexes. We discovered 5 protein modules having 3 or more members (Fig. 5A–E), with no statistically significant functional enrichment, expected because transcriptional networks do not necessarily correspond to protein-protein interaction networks. We therefore queried integrated protein-protein interactome databases for first-degree neighbors of each of these module members, thus expanding the respective modules (Fig. 5F–J). These first degree expanded protein modules were statistically significantly enriched for RNA-dependent DNA replication/telomere maintenance via telomerase (B-H corrected *P* = 7.83 × 10^−3^) (Fig. 5F); ligand dependent transcription factor activity (B-H corrected *P* = 3.43 × 10^−2^) (Fig. 5G); Tumor Necrosis Factor (TNF) binding (B-H corrected *P* = 6.95 × 10^−4^) and p53 mediated DNA damage response (B-H corrected *P* = 2.47 × 10^−2^) (Fig. 5H); basal lamella (B-H corrected *P* = 1.68 × 10^−10^) and substrate dependent cell movement (B-H corrected *P* = 6.28 × 10^−4^) (Fig. 5I); and actin filament sliding (B-H corrected *P* = 8.22 × 10^−3^) and endocytosis (B-H corrected *P* = 2.06 × 10^−2^) (Fig. 5J).

### Methyl CpG islands associated with repeated DNA elements

**Figure 6:**
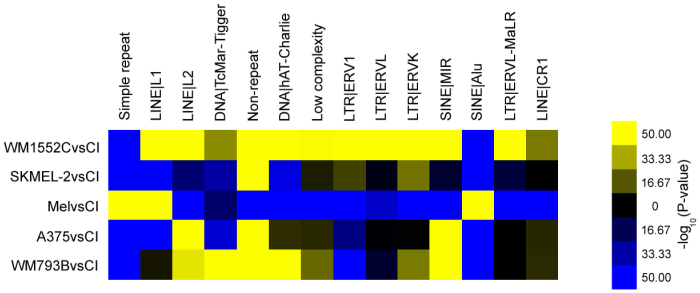
Patterns of methyl CpG at repetitive elements enriched in the five cell lines. The relative levels of MBD2-enrichment at repeated DNA elements compared to input (un-enriched) DNA are plotted in a color coding scheme in which the color represents the enrichment significance P-values.

Given that one of the expanded protein modules discovered above is enriched for RNA-dependent DNA replication, and the suspected role of retrotransposable elements in causing large-scale genomic rearrangements in cancer, we looked for the evidence of altered CpG island methylation associated with repeated DNA elements in melanoma cell lines and compared these against the melanocyte derived cell line (HEM-l). Among the 14 classes of repeated elements we tracked, 11 classes were associated with relatively hypomethylated CpG islands in late stage melanomas (WM1552C, SK-MEL2, and A375) compared to their methylation status in melanocytes (Fig. 6). The methylation levels of the CpG islands associated with 7 of these 11 elements appeared relatively unchanged in the remaining melanoma cell lines including the Stage I line WM793B, the remaining four showing a slight bias towards hypomethylation. Strikingly, three repeat element classes (simple repeats, LINE-1 elements, and SINE Alu repeats) showed the opposite tendency: hypermethylation in WM1552C, SK-MEL2 and A375 cells relative to HEM-l and WM793B. While these results are consistent with the notion that some transposable elements characterized by these repeat elements could become active due to hypomethylation in late stage melanomas, there is variation with respect to the classes of moveable elements as well as the exact stage of melanoma development. In general, it appears that hypomethylated repeat elements are more frequent in advanced stage melanomas than in stage I or in normal melanocytes.

### Methylated CpG islands show consistent signatures in melanoma patient samples

To address whether the general patterns of CpG island methylation discovered above are reflected in frozen tumor tissue samples obtained from malignant melanoma (stages III and IV) patients, we measured CpG island methylation status of upstream regulatory regions of SNORD-10 (Fig. 4B) and c-KIT (Fig. S5). Unlike the unmethylated CpG islands in non-cancerous nevi samples, all tumor samples showed variable levels of hyper-methylated CpG islands. These results suggest that the set of 581 upstream regulatory regions discovered here as differentially methylated among melanoma cells provide a novel collection of regions for further subtyping of melanoma cells in future studies. Muthusamy et al., has previously reported that several coding genes promoter CpG islands, including PCSK1, QPCT and CYP1B1 have been methylated in melanoma patient samples and our cell line results agree with their observations^53^.

## Discussion

We have determined by deep sequencing the genome wide distribution of MBD2-pulldown DNA from one normal melanocyte cell line and four melanoma cell lines, and by targeted bisulfite sequencing have demonstrated that the CpG islands in the MBD2-affinity captured DNA are enriched for methylated CpG residues. A notable feature of the results from the stage III and IV melanoma samples is that CpG islands shorter than ~ 270 bp were proportionately more hypermethylated than the longer (> 450 bp) CpG islands when compared with the methylation levels of the corresponding CpG stretches of normal melanocytes and the stage I melanoma lines. We have identified a core group of 19,686 CpG island sequences that mapped to at least 821 genes or their associated regions. The distribution of the methyl CpG residues within various genic and non-genic features exhibit statistically significant hypermethylation in the 3 kb upstream promoter regions, introns and exons in stage III and IV melanoma cell lines relative to the corresponding features in the normal melanocyte cells. Recently, Kim et al.^70^ reported that the intronic regions are highly methylated in prostate cancer; similarly, in our dataset the highest levels of methyl-CpGs were also associated within intronic regions, and these regions were equally methylated among all cell lines, which presumably reflect transcriptionally an inert state of these DNA stretches. Hansen et al.^71^ previously reported a study focus on methylated DNA regions in several cancer types (colon, lung, breast, thyroid and wilm's tumors), but not in melanoma.

A large fraction of the hypermethylated CpG islands in the stage III cell line WM1552C could be erased by treating these cells with 5AzadC. While there are significant hypermethylation of CpG islands associated with genes and their surrounding regions in the advanced stage melanoma cell lines, the same cell lines show significantly reduced CpG methylation levels associated with LINE1, LINE2 and various LTR repeat elements. Our observations indicate that while there is evidence of extensive hypermethylation of genic and non-genic regions in stage III–IV melanoma cell lines, the reverse is true of the CpG islands associated with 11 out of 14 types of repetitive DNA elements. Most of these repetitive retro-elements are hypomethylated in the stage III–IV groups. Since hypomethylation of retro-element DNA is known to positively correlate with their movement, and because previous reports of genome wide studies have shown increased levels of mutagenesis in melanoma cell lines, we suggest that a significant proportion of genome wide DNA sequence alterations in late stage melanoma cells could be due to retro-element mobilization. However, there are subtle differences in the behavior of CpG islands associated with retro-element classes. For example, the CpG islands associated with SINE L1 elements show the reverse tendencies (are hypermethylated) relative to SINE L2 elements; similarly, SINE Alu repeat-associated CpG islands are hypermethylated in the stage III–IV group compared with the SINE MIR elements. It will be interesting to determine whether the relative mobility of these elements reflects their corresponding CpG island methylation patterns.

Among the genes that are differentially methylated at CpG islands within 1 kb upstream of their TSS, which are also differentially expressed in the stage III melanoma line WM1552C relative to those in normal melanocytes, we have identified a group of 554 genes that appear to be co-expressed in a large dataset of cancer-related gene expression levels. This group of genes is functionally enriched for transcription factors, and we suspect that a significant proportion of these genes might form a network of epigenetic core that are coordinately regulated by methylation at their CpG islands. Interestingly, this co-expression network is also enriched for alpha-2 macroglobulins, which are known to be over-expressed in melanoma cells^72^,^73^,^74^, which may be related to their invasiveness and metastasis^72^. Strikingly, this large co-expression network does not appear to be enriched for members that encode proteins that are part of protein complexes. However, by systematically querying the current protein-protein interaction databases, we were able to discover the memberships of several of these proteins within five functionally significant protein complexes: RNA-dependent DNA polymerases, transcription factors, DNA damage/cell death response, cell motility, and basal lamella function. The first of these complexes are known to be important for retrotranspose on mobility, and the remaining categories of proteins are all known to be associated with advanced stage cancers including metastatic cancers. Noteworthy also is the inclusion of laminin, which is a known mitogen for melanoma cells^75^.

The genome wide data on methylated CpG island regions enriched in late state melanoma cells relative to melanocytes described here constitute a resource for cancer biologists and melanoma researchers. Investigation of the epigenetic control of human melanomas should contribute to molecular classification of metastatic melanomas; the resources described here should be useful for these studies. We suggest that the CpG island methylation signatures revealed here should be important for the development of novel biomarkers, as for example we have shown a remarkably consistent methyl CpG signature at the noncoding RNA gene SNORD-10 by direct bisulfite sequencing selected on the basis of our genome wide data. However, the development of such biomarkers would have to depend on further retrospective and prospective studies on additional patient samples.

In conclusion, we have described genome-wide distribution of methylated CpG islands in a number of melanoma cell lines, and have shown that these regions are functionally significant for cancerous properties of these cells.

## Methods

### Cell lines and clinical samples

The experiments performed in this manuscript utilized the following cells and cell lines: human epidermal melanocyte cell line HEM-l (acquired from ScienCell, Catalog # 2200; growth conditions: MelM media containing MelGS supplements with 0.5% FBS and pen/strep), the melanoma cell lines WM793B (stage 1, ATCC® Number: CRL-2806), WM1552C (stage 3, ATCC® Number: CRL-2808), SK-MEL2 (stage 4, ATCC(R)), and A375 (stage 4, ATCC® Number: CRL-1619). All melanoma cell lines were grown in Complete Tu media: a 4:1 mixture of MCDB-153 w/1.5 g/L sodium bicarbonate & Leibovitz L-15 with 2 mM L-glutamine, plus 2% FBS and 1.68 mM CaCl2. All clinical samples described in this publication were acquired from frozen samples graciously donated by Dr. James Goydos of Robert Wood Johnson Medical School^76^.

### MBD2 pull-down

Genomic DNA was fragmented to 50–400 bp (mean ~ 250 bp) using the Covaris™ S2 system (Woburn, MA), and 10 μg of DNA was subjected to MBD2-protein capture according to the manufacture's protocol. We used different concentrations of NaCl enrichment buffer (200 nM, 350 nM, 450 nM, 600 nM, 1 M, and 2 M) for single fraction elution in A375 cells and observed that the highest salt concentration (2 M) produced tighter enrichment of CpG islands in the MBD2 pull-down samples. Therefore, we used 2 M NaCl for all subsequent sample enrichments. The methylated DNA fragments recovered from the first two incubations of 2 M salt were pooled, and then ethanol precipitated and resuspended. This DNA or samples of DNA that did not go through the MBD2 pull-down (whole-genome, non-enriched), were used for fragment library construction, which included a gel-based size selection step to obtain a mean fragment length of ~ 150 bp.

### Isolation of genomic DNA and bisulfite treatment

Samples of genomic DNA were prepared from 10^7^ cells of each cell line. Samples were harvested by trypsinization, spun down at low speed (1200 rpm), and washed with phosphate-buffered saline (PBS). Cell pellets were then processed using the QiaAmp DNA mini kit (QIAGEN). Genomic DNA was harvested from 25 mg of frozen patient samples and isolated by incubation with proteinase K at 55°C overnight. Samples were purified with the QiaAmp DNA mini kit (QIAGEN). All sample yields were determined using a ND-1000 spectrophotometer (Nanodrop). 500 ng of each sample of genomic DNA was then treated with sodium bisulfite overnight and purified and eluted with the EZ DNA methylation kit (Zymo Research).

### Bisulfite-conversion and amplification

Bisulphite PCR was performed using genomic eluate with the following primer combinations: PCSK1 Meth For (gggtagataaggagtagatttaattgattttag) and PCSK1 Meth Rev (ctctaaaccactcctaactcctaattactc) to amplify a 253 bp region; CYP1B1 Meth For 2 (ggagttgattttttggagaaatggt) and CYP1B1 Meth Rev (cttaccctaaacaaaaatcccaattccttc) to amplify a 301 bp region; QPCT Meth For (gggtttagaagtttgtgtttgttatttaggg) and QPCT Meth Rev 3 (cccaaaacaaaacgaccaccaacaacaac) to amplify a 243 bp region; TERC Meth For (gggttagtagttgatattttttgtttgttttag) and TERC Meth Rev 2 (cctaaaaaaaataataaccattttttatctaaccc) to amplify a 107 bp region; Kit Meth For (tgggaggaggggttgttgtt) and Kit Meth Rev 2 (taccaccctcccaaacacaaacttc) to amplify a 210 bp region; and SNORD 10 Meth For (ggtggttatggtattaggagattatatggg) and SNORD 10 Meth Rev (ctcttcccccaaaaaaaaaccaacatcc) to amplify a 219 bp region. All PCRs reactions were performed using a 2-min hot start at 95°C, followed by 35–40 cycles at 94°C for 20 s, 54°C for 20 s, and 72°C for 45 s, ending with a 2-min extension at 72°C using GoTaq Green (Promega). PCR products were gel purified from agarose gels using QiaQuick gel extraction kit (QIAGEN) and subsequently cloned into the pCR4-TOPO vector (Invitrogen/Life Technologies). Six independent clones for each target region were sequenced (Retrogen) using the primers M13 For (−20) and M13 Rev. Sequences were then aligned and analyzed using VectorNTi AlignX (Invitrogen/Life Technologies).

### Treatment of melanoma cells with 5-aza-2'-deoxycytidine (5AzadC)

Stage III melanoma cells (WM1552C) were plated into 75-cm^2^ flasks at a concentration of 5 × 10^5^ cells per flask. Each flask was treated with 10 μg/mL 5AzadC, with a control flask left untreated. The cells were then washed daily with PBS, given fresh media, and treated as described above. Treatment was continued for 5 days after which the cells were washed again with PBS, trypsinized and harvested, and centrifuged at 1200 rpm for 5 min. Total RNA was extracted from the cell pellets with Trizol reagent (Invitrogen/Life Technologies), and quantified using the ND-1000 spectrophotometer (NanoDrop). Each assay was performed in triplicate.

### High-throughput sequencing

Methylated regions were enriched using LifeTech MethylMiner™ Methylated DNA Enrichment kit (LifeTech, Foster City, CA). The SOLiD™ System Fragment Library Preparation protocol was followed per the manufacturer's instructions (LifeTech, Foster City, CA, USA). Emulsion PCR (ePCR) was performed according to standard LifeTech SOLiD™ 3.0 System. Templated beads were deposited onto two slide quadrants per sample and sequencing was carried out to 50 bases using SOLiD™ v3.0 chemistry and the manufacturer's instructions.

### Sequence data processing

The human genome NCBI Build 37/hg19 (downloaded from UCSC) was used for the mapping and data analysis. The raw data from SOLiD™ 3.0 instrument were pre-processed using SETS software (v3.5, LifeTech, Foster City, CA) for image analysis, color calling and run quality metrics respectively. Then the color-space raw reads were mapped to human genome using Bowtie aligner (v0.12.5)^77^, allowing a maximum of three mismatches per reads. Only uniquely mapped reads were retained for further data analysis. For each sample, the aligned reads from two technical replicates were pooled together to provide greater coverage for identifying the potential methylation regions in this study.

### Identification of methylated regions

Each MBD2-pulldown and its corresponding input sample DNA was sequenced as above. The uniquely mapped reads were processed using MACS 1.4 for peak identification^78^. The enriched methylated regions were called by comparing the pooled samples with the input control, with a p-value of less than 1.0E-10 as cutoff using Poisson distribution model in MACS. All methylation regions were converted into wiggle format for downstream data analysis and visualization in UCSC genome browser. The Circos software^79^ was applied to visualize the genome-wide distribution of the methylation patterns.

### RNA-sequencing and data analysis

Total RNA was obtained from melanocyte, WM1552C and 5AzadC treated WM1552C cell lines using standard RNA isolation techniques, and fragmented by RNAaseIII digestion. Reverse transcription of these RNAs will be primed from a ligated primer, and the resulting cRNA will be amplified and size-selected (150 ~ 250 bp) for sequencing on a 6% urea gel with the help of SYBR Gold dye. Sequencing library preparation and emulsion PCR were performed based on standard LifeTech protocols (LifeTech, Foster City, CA, USA), and short read sequencing was carried out to 50 bases using SOLiD™ v3.0 chemistry.

The raw RNA-seq data were aligned to hg19 reference genome using Bowtie aligner (v0.12.5)^77^, and TopHat^80^. The mapping results were converted into big wiggle format and visualized in same UCSC genome browser session as methylation data. RNA-seq quantification and differential expression analysis were performed used RNA-seq workflow in the Partek Genomics Suite™ (version 6.5, Partek Inc, St. Louis, MO). Briefly, the Partek workflow summarized the transcript abundance based on RefSeq transcripts using Expectation-Maximization (EM) approach. The reads per kilobase of transcript per million mapped sequence reads (RPKM) were computed, and ANOVA was applied for transcript-level differential expression detection. Transcripts with RPKM less than 1 in all the study samples were filtered out from further analysis.

### Estimating size distribution of the methylated CpG islands

The CpG islands were downloaded both from UCSC Genome Browser (version hg19, 28,691 islands), and the newly defined CpG islands based on a Hidden Markov Model^81^ (http://rafalab.jhsph.edu/CGI/, 65,699 islands). The final CpG islands (72,145 islands) were generated from a union of the two annotations. To determine the size distributions, we accepted the methylated regions identified by MACS as potential methylated CpG islands, and the frequency for each length class was computed by normalizing the number of methylated CpG islands exhibiting this length by the total number of methylated CpG islands identified.

### Association of CpG islands with extraneous features

To assess the potential functions of methylated CpG islands, we extended each island boundary by 200 bp in both upstream and downstream directions and looked for overlaps with other features such as lncRNA, promoter, gene structure, etc. Genomic regions were defined based on NCBI Build 37/hg19 coordinates downloaded from the UCSC web site. Long noncoding RNA annotations were identified from a pool of the human GenCode v12^82^ and the UCSC Human “All mRNA” transcripts^83^ by a process of exclusion (i.e. those transcripts that cannot be defined as protein-coding are considered to be noncoding). Protein-coding transcripts were classified if they met any of the following criteria: (1) any exon overlapped by 1 bp or more with the exon (CDS or UTR) of a GenCode protein-coding gene; (2) were defined as protein-coding using Coding Potential Calculator^84^; or (3) contained an open reading frame greater than 120 codons that comprised more than one third of the transcript length. All remaining transcripts were classified as noncoding. In addition the protein-coding classification was overridden if the transcript was annotated in lncRNAdb^85^.

### Hierarchical clustering of methylminer data

For this analysis the regulatory region was defined as a 2000 bp window centered around each TSS (http://dbtss.hgc.jp/). We chose only the cancer related genes extracted from the CancerGene^66^ database. Genes whose regulatory regions showed no methylation in all five cell lines were excluded; 3,688 genes remained after this filtering. We estimated the methylation level of each gene's regulatory region in a specific cell line using the number of reads that have been mapped within that region. The number of reads was normalized into effective reads by equating the total number of reads per cell line to the total number of reads in the input control. The fold change for each gene's regulatory region in each cell line was computed as the ratio of the effective read at this region of the sample to that of the input/control. The fold change for each gene across different cell lines was further normalized such that the fold change had a mean of 0 and standard deviation of ± 1.0. Dendrograms were created using hierarchical clustering implemented in MATLAB using Pearson's correlation coefficient method.

### Other computational and statistical methods

GO functional enrichment analysis of the CpG-associated genes was conducted by the Ingenuity Pathway Analysis software suite (IPA Ingenuity Systems, www.ingenuity.com). Fisher's Exact Test P-values were adjusted for false discovery rate by the Benjamini-Hochberg correction with P cut-off at 0.05 (or –log P > 1.3). Gene co-expression network was constructed by querying the integrated gene interaction database GeneMANIA^86^; GO enrichment of the connected network component was measured using GOStat^87^ (http://gostat.wehi.edu.au/); protein-protein interaction networks were constructed by querying GeneMANIA and all network data were analyzed using Cytoscape^88^. Student's t-test and Fisher's Exact test were also performed to compute the significant level of differential distribution of methylated using MATLAB.

## Author Contributions

J.L.L., A.R., S.Z. and R.J.P. were involved in the initial planning of the experiment. J.L.L., C.Z., G.J.F., Z.Z., S.S.G., C.A., G.M. and M.E.D. were involved in experiment design, next-generation DNA sequencing, statistics, sequence data analysis and bioinformatics. J.M. carried out the molecular biology and molecular genetics analysis. J.S.M., A.R., R.J.P. wrote the manuscript. All authors revised the manuscript.

## Supplementary Material

Supplementary Information Supplementary Information
