# Supplementary material for: Genome-wide methylated CpG island profiles of melanoma cells reveal a melanoma coregulation network
Source: Sci Rep. 2013 Oct 16;3:2962. doi: 10.1038/srep02962 (PMC3797435; doi:10.1038/srep02962)
Supplement: Supplementary Information [file srep02962-s1.pdf]

Supporting Information

Genome-wide methylated CpG island profiles of melanoma cells reveal a melanoma coregulation network

Jian-Liang Li[1], Joseph Mazar[1†], Cuncong Zhong[2†], Geoffrey J Faulkner[3,4], Subramaniam S Govindarajan[1], Zhan Zhang[1], Marcel E Dinger[5], Gavin Meredith[6], Christopher Adams[6], Shaojie Zhang[2], John S Mattick[5], Animesh Ray[7*], and Ranjan J Perera[1*]

[1]Sanford-Burnham Medical Research Institute, Orlando FL 32827 USA

[2]Department of Electrical Engineering and Computer Science, University of Central Florida, Orlando FL 32816 USA

[3]Cancer Biology Program, Mater Medical Research Institute, South Brisbane, Queensland 4101, Australia

[4]School of Biomedical Sciences, University of Queensland, Brisbane, Queensland 4072, Australia

[5]Garvan Institute of Medical Research, Darlinghurst NSW 2010, Australia

[6]Life Technologies, Carlsbad CA 92008 USA

[7]School of Applied Life Sciences, Keck Graduate Institute, Claremont CA 91711 USA

[†]These authors contributed equally to this work

*Corresponding authors. rperera@sanfordburnham.org / Animesh_Ray@kgi.edu

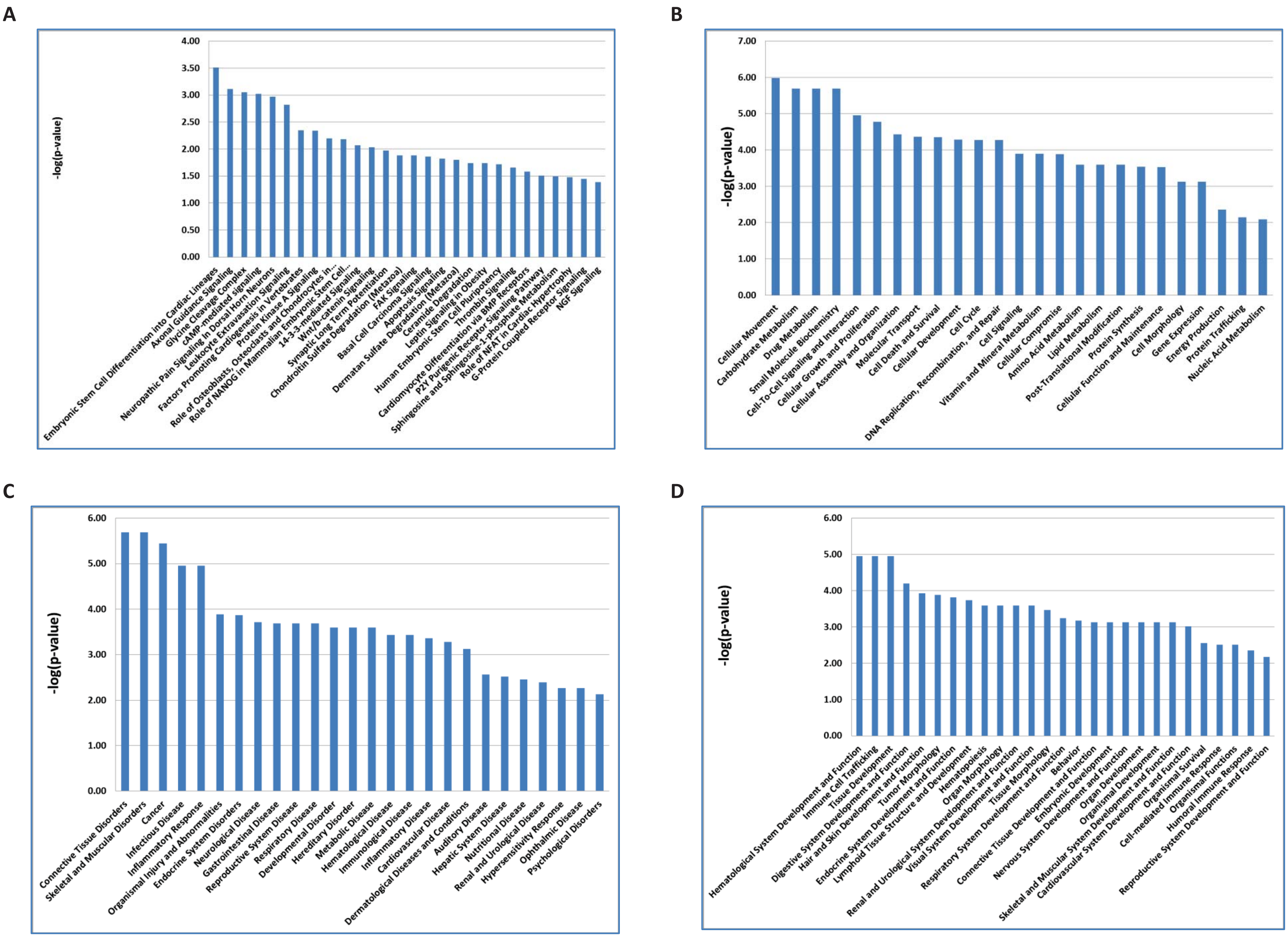

Figure S1 - Related to Figure 1 E.

Functional enrichment analysis for 821 genes whose upstream regulatory regions were highly methylated only in two Stage IV cell lines. A. Canonical Pathways; B. Molecular and Cellular Functions; C. Diseases and disorder; D. Physiological System Development and Function.

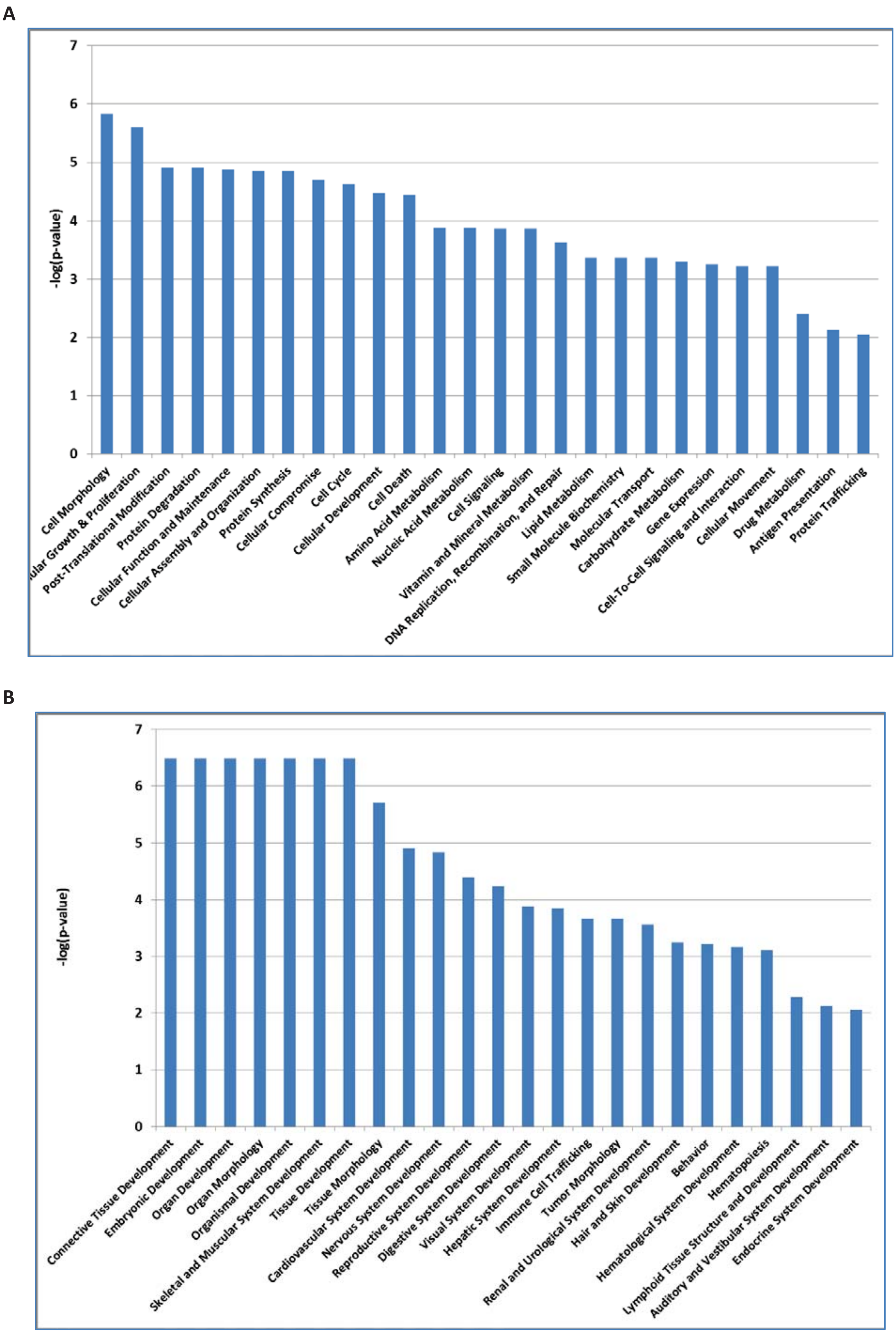

Figure S2 - Related to Figure 3

Functional classification for 581 genes whose upstream regulatory regions were highly methylated. A. Molecular and Cellular Functions; B. Physiological System Development and Function.

A

B

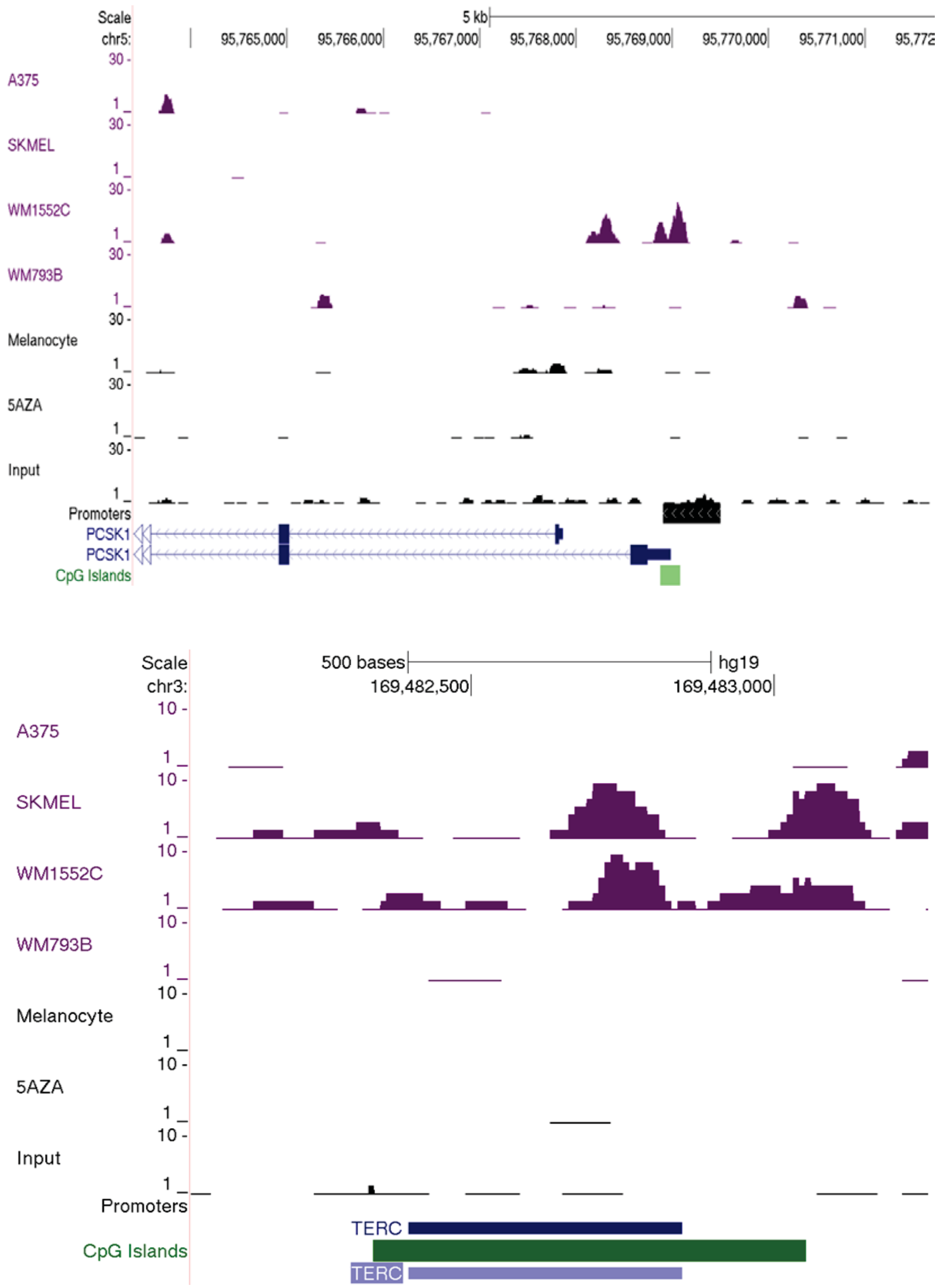

Figure S3 - Methylation regions in two example genes cross different cell lines.
A) The CpG islands in the upstream of PCSK1 were methylated in WM1552C but not in any of the other tested cell lines. B) The upstream putative promoter regions of noncoding TERC are highly methylated at the CpG islands and are revered upon treatment with 5Azadc.

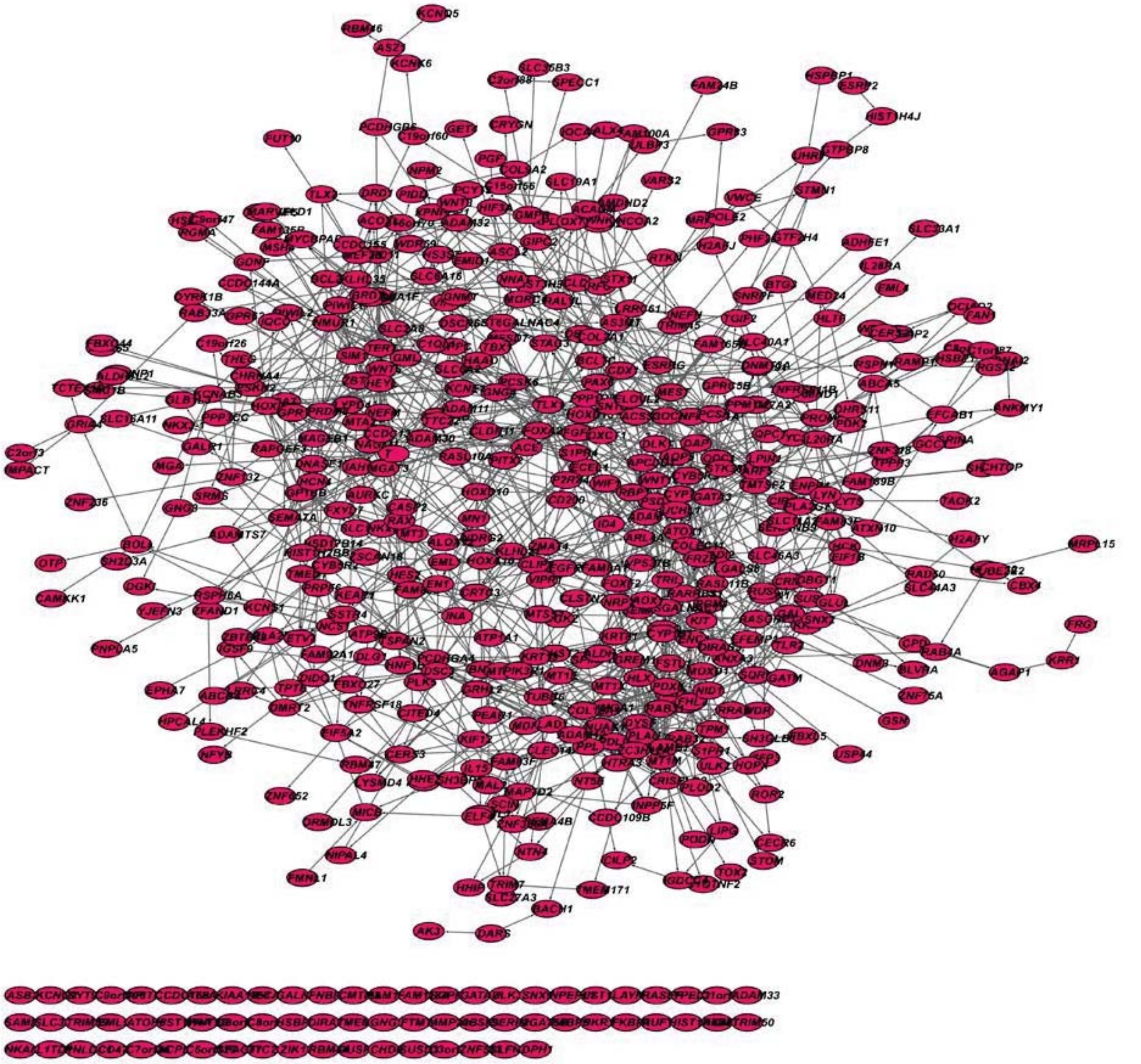

Figure S4 - Related to Figure 5

MED2-enriched DNA from WM1552C cells defines a large co-expression network with cancer and metastasis-related genes.

A co-expression network formed by the genes that exhibit (a) enrichment for MBD2-pulldown relative to HEM-1, (b) differential RNA expression levels measured by RNA-seq relative to HEM-1, and (c) included in cancer-related genes by Higgins et al [66]. Of the 581 genes in this set that meet all three criteria above, 554 genes are in the connected component. The network fits a power law degree distribution and is enriched for sequence-specific DNA binding transcription factors (see text).

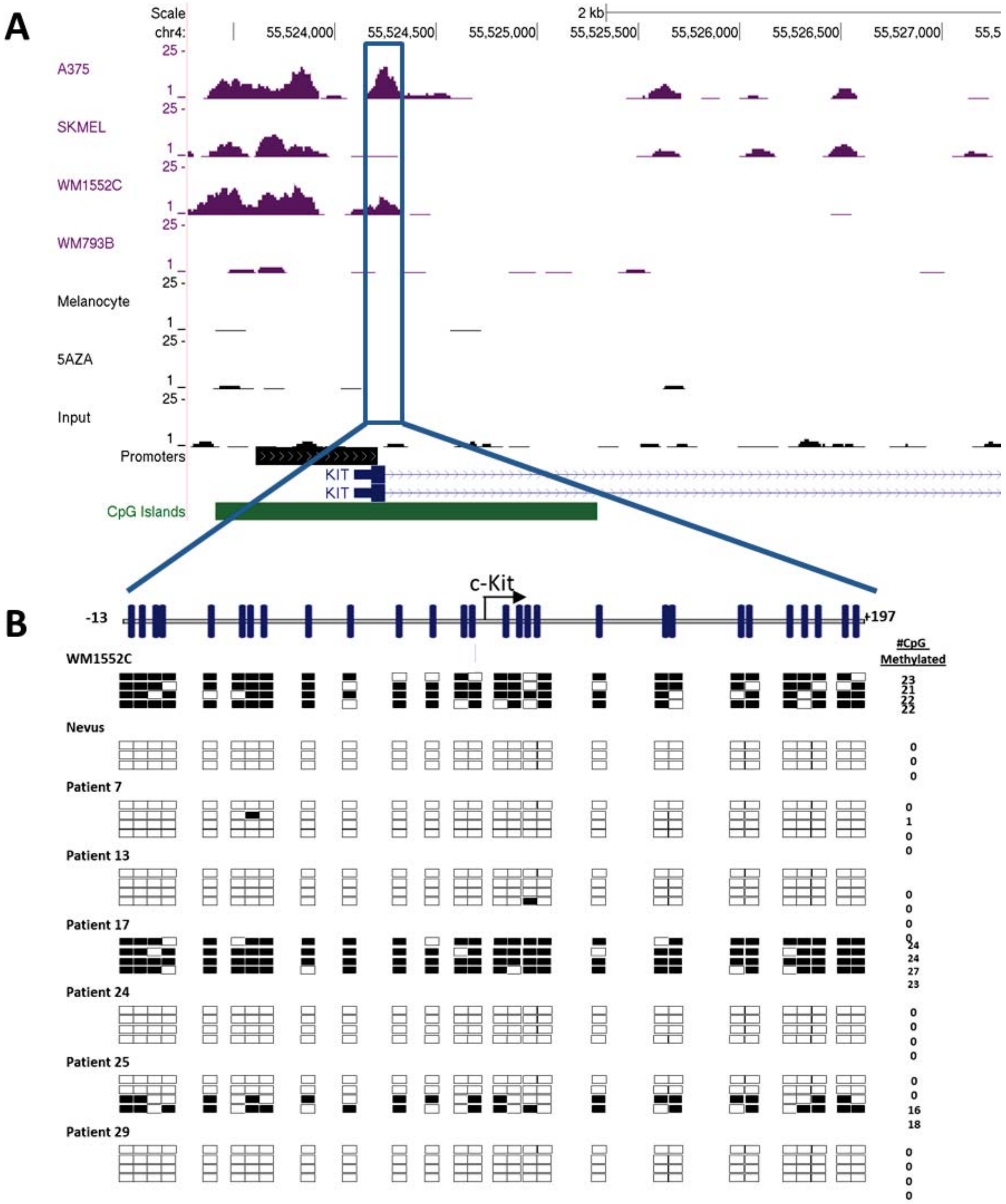

Figure S5 - CpG methylation status near c-KIT gene in different patient samples
(A) depicts the results obtained with NextGen sequencing of MBD2-enriched DNA in the five cell lines, plus those in WM1552C cells treated with 5AzadC and those in the input DNA. (B) A region between -13bp and +197bp of the TSS of c-KIT is shown magnified. Methylation status of this region in the DNA obtained from various metastatic melanoma patient samples or in non-melanoma nevi was determined by bisulfite sequencing (see Methods). Each box is a CpG; open boxes are unmethylated, filled boxes are methyl CpG sequences. Numbers at the right represent the number of methyl CpG detected in the region corresponding to each row (independent sequence analysis).

Table S1

Summary of sequencing data results (Related to Figure 1a)

Cell Line	Stage	Total Read	Unique mapped read	Percent of total reads	Coverage[a]	Peak calling regions	Regions closed to genes[b]	Closest Genes
Melanocytes		59,597,072	16,053,227	26.94%	20.07	145,221	90,140	14,998
WM793B	Stage 1	71,857,478	14,116,776	19.65%	17.65	113,657	65,001	12,762
WM1552C	Stage 3	68,960,602	21,988,850	31.89%	27.49	284,533	193,148	16,264
A375	Stage 4	70,076,583	23,148,948	33.03%	28.94	216,021	150,254	15,648
SK-MEL2	Stage 4	66,018,932	18,760,175	28.42%	23.45	253,796	190,083	15,241

a. Assuming 1~2% of human genome are CpG clusters which were pull down by MBD2. The coverage calculation was based on the Lander and Waterman equation.
b. The enriched regions are located between 3K upstream of TSS and 3K downstream of TES.

Table S2

Fisher's exact test significant p-value for genome-wide distribution of differential methylated regions between cell lines. P-values in bold and italics indicate significant difference (≤ 0.05). (Related to Figure 3A)

Comparison	Promoter 3K	Downstream 3K	5' UTR	3' UTR	Exon	Intron
WM793B vs Melanocytes	0.1119	0.6526	0.3744	0.1464	***2.38E-20***	***2.39E-04***
WM1552C vs Melanocytes	1.0000	0.7178	1.0000	0.6479	0.5747	0.3942
SK-MEL2 vs Melanocytes	0.6082	0.9273	1.0000	0.7582	0.5596	0.7042
A375 vs Melanocytes	0.8383	0.7178	0.7533	0.5469	0.4730	0.4240
WM1552C vs WM793B	0.1119	1.0000	0.3744	***0.0392***	***9.47E-23***	***4.75E-06***
SK-MEL2 vs WM793B	***0.0287***	0.7216	0.3744	0.0555	***2.83E-18***	***9.88E-04***
A375 vs WM793B	0.0583	0.9293	0.1243	***0.0274***	***2.35E-23***	***6.10E-06***
SK-MEL2 vs WM1552C	0.6082	0.7886	1.0000	0.8840	0.2214	0.1991
A375 vs WM1552C	0.8383	1.0000	0.7533	1.0000	0.9377	0.9580
A375 vs SK-MEL2	0.8351	0.8576	0.7532	0.8840	0.1931	0.2382

Table S3. 581 candidate genes with RNA-seq abundance results.

List of 581 candidate genes whose the CpG island in the regulatory regions was methylated. The corresponding RNA-seq expression data were also presented in the table.

Note:

a. Melanocyte cell line against WM1552C cell line
b. WM1552C with 5AZA against WM1552C cell line
c. ND: non detectable. The RPKM for both cell lines are less than one
d. -∞: non reads in Melanocyte cell line

Methylated Region	Gene Symbol	Gene Description	Fold change[a] (Mel/WM1552C)	Fold change[b] (5AZA/WM1552C)
chr1:1141874-1142398	TNFRSF18	tumor necrosis factor receptor superfamily, member 18	ND[c]	ND
chr1:1630935-1631151	MMP23A	matrix metallopeptidase 23A (pseudogene)	ND	ND
chr1:6480289-6481218	HES2	hairy and enhancer of split 2 (Drosophila)	ND	ND
chr1:6639329-6639723	ZBTB48	zinc finger and BTB domain containing 48	-5.08	2.38
chr1:6663606-6663943	KLHL21	kelch-like 21 (Drosophila)	-5.23	2.38
chr1:10489950-10490274	APITD1	apoptosis-inducing, TAF9-like domain 1	ND	1.62
chr1:11713970-11714468	FBXO44	F-box protein 44	ND	ND
chr1:17445996-17446624	PADI2	peptidyl arginine deiminase, type II	ND	ND
chr1:19600569-19600922	AKR7L	aldo-keto reductase family 7-like	ND	ND
chr1:24514310-24514819	IL28RA	interleukin 28 receptor, alpha (interferon, lambda receptor)	ND	ND
chr1:26233169-26233793	STMN1	stathmin 1	-2.42	-2.91

chr1:32670484-32670965	IQCC	IQ motif containing C	ND	ND
chr1:37939662-37939933	ZC3H12A	zinc finger CCCH-type containing 12A	-33.30	-1.18
chr1:40105460-40105957	HEYL	hairy/enhancer-of-split related with YRPW motif-like	ND	ND
chr1:40157572-40158395	HPCAL4	hippocalcin like 4	ND	5.13
chr1:40783072-40783567	COL9A2	collagen, type IX, alpha 2	ND	ND
chr1:41328232-41328576	CITED4	Cbp/p300-interacting transactivator, with Glu/Asp-rich carboxy-terminal domain, 4	ND	ND
chr1:41444329-41444938	CTPS1	CTP synthase 1	-16.63	-1.38
chr1:47134182-47134474	ATPAF1	ATP synthase mitochondrial F1 complex assembly factor 1	ND	ND
chr1:53526999-53527727	PODN	podocan	ND	ND
chr1:55266944-55267363	TTC22	tetratricopeptide repeat domain 22	ND	ND
chr1:60539203-60540202	C1orf87	chromosome 1 open reading frame 87	ND	ND
chr1:62660116-62660651	L1TD1	LINE-1 type transposase domain containing 1	ND	ND
chr1:64058483-64058915	PGM1	phosphoglucomutase 1	-3.64	1.20
chr1:67217514-67218603	TCTEX1D1	Tctex1 domain containing 1	ND	ND
chr1:68517067-68517577	DIRAS3	DIRAS family, GTP-binding RAS-like 3	ND	ND
chr1:76189660-76189980	ACADM	acyl-CoA dehydrogenase, C-4 to C-12 straight chain	-3.21	1.21
chr1:76262420-76263058	MSH4	mutS homolog 4 (E. coli)	ND	ND
chr1:78511174-78512557	GIPC2	GIPC PDZ domain containing family, member 2	ND	ND
chr1:87169359-87170016	SH3GLB1	SH3-domain GRB2-like endophilin B1	-2.83	1.59
chr1:92414408-92415142	BRDT	bromodomain, testis-specific	ND	ND
chr1:95285136-95285615	SLC44A3	solute carrier family 44, member 3	ND	ND
chr1:101701691-101702360	S1PR1	sphingosine-1-phosphate receptor 1	ND	1.63
chr1:115632219-115632830	TSPAN2	tetraspanin 2	ND	8.46

chr1:116914993-116915634	ATP1A1	ATPase, Na+/K+ transporting, alpha 1 polypeptide	-1.44	3.14
chr1:117664942-117665496	TRIM45	tripartite motif containing 45	ND	ND
chr1:118727490-118728191	SPAG17	sperm associated antigen 17	ND	ND
chr1:120438613-120439341	ADAM30	ADAM metallopeptidase domain 30	ND	ND
chr1:150947661-150948469	CERS2	ceramide synthase 2	-4.57	1.98
chr1:153605662-153606369	CHTOP	chromatin target of PRMT1	-4.84	2.26
chr1:153747386-153747896	SLC27A3	solute carrier family 27 (fatty acid transporter), member 3	ND	3.27
chr1:155225418-155226249	FAM189B	family with sequence similarity 189, member B	ND	ND
chr1:155290146-155291079	RUSC1	RUN and SH3 domain containing 1	ND	ND
chr1:156862435-156863796	PEAR1	platelet endothelial aggregation receptor 1	ND	ND
chr1:159915685-159915852	IGSF9	immunoglobulin superfamily, member 9	ND	ND
chr1:161171285-161171854	NDUFS2	NADH dehydrogenase (ubiquinone) Fe-S protein 2, 49kDa (NADH-coenzyme Q reductase)	-6.43	1.49
chr1:171809795-171810544	DNM3	dynamin 3	ND	ND
chr1:178510988-178511324	C1orf220	chromosome 1 open reading frame 220	$-\infty^{d}$	-1.23
chr1:181057233-181057680	IER5	immediate early response 5	-10.21	3.49
chr1:182361238-182361652	GLUL	glutamate-ammonia ligase	ND	ND
chr1:201368833-201369125	LAD1	ladinin 1	ND	ND
chr1:217263138-217263909	ESRRG	estrogen-related receptor gamma	ND	ND
chr1:221051530-221052508	HLX	H2.0-like homeobox	ND	ND
chr1:228612976-228613341	HIST3H3	histone cluster 3, H3	ND	ND
chr1:229406104-229406760	RAB4A	RAB4A, member RAS oncogene family	-4.22	1.35
chr1:235813960-235814580	GNG4	guanine nucleotide binding protein (G protein), gamma 4	ND	ND
chr1:236228661-236229395	NID1	nidogen 1	-26.65	-6.72

chr1:236686471-236687097	LGALS8	lectin, galactoside-binding, soluble, 8	-7.35	1.93
chr10:8095586-8096352	FLJ45983	uncharacterized LOC399717	ND	ND
chr10:8096511-8096778	GATA3	GATA binding protein 3	ND	ND
chr10:73723287-73723692	CHST3	carbohydrate (chondroitin 6) sulfotransferase 3	-13.20	-1.20
chr10:75670267-75670910	PLAU	plasminogen activator, urokinase	ND	ND
chr10:92913010-92913794	NUDT9P1	nudix (nucleoside diphosphate linked moiety X)-type motif 9 pseudogene 1	ND	ND
chr10:93392398-93393705	PPP1R3C	protein phosphatase 1, regulatory subunit 3C	638.23	ND
chr10:94448455-94449365	HHEX	hematopoietically expressed homeobox	-84.36	-4.14
chr10:97051010-97051451	PDLIM1	PDZ and LIM domain 1	-21.83	1.26
chr10:99472630-99473110	MARVELD1	MARVEL domain containing 1	-1.75	-1.09
chr10:102890802-102891269	TLX1	T-cell leukemia homeobox 1	ND	ND
chr10:104628754-104629225	AS3MT	arsenic (+3 oxidation state) methyltransferase	4.99	ND
chr10:105036152-105036801	INA	internexin neuronal intermediate filament protein, alpha	ND	ND
chr10:111683538-111683877	XPNPEP1	X-prolyl aminopeptidase (aminopeptidase P) 1, soluble	-5.21	1.95
chr10:121578298-121578731	INPP5F	inositol polyphosphate-5-phosphatase F	ND	1.59
chr10:124638964-124639376	FAM24B	family with sequence similarity 24, member B	ND	1.90
chr10:128077333-128077666	ADAM12	ADAM metallopeptidase domain 12	ND	1.35
chr11:804110-804955	PIDD	p53-induced death domain protein	-8.60	2.93
chr11:2292673-2293299	ASCL2	achaete-scute complex homolog 2 (Drosophila)	ND	ND
chr11:2890161-2890757	KCNQ1DN	KCNQ1 downstream neighbor (non-protein coding)	ND	ND

chr11:3239065-3239647	MRGPRG-AS1	MRGPRG antisense RNA 1	ND	ND
chr11:7272678-7273091	SYT9	synaptotagmin IX	ND	ND
chr11:7695089-7695915	CYB5R2	cytochrome b5 reductase 2	-20.74	-1.27
chr11:31832659-31833171	PAX6	paired box 6	ND	ND
chr11:33036608-33037208	DEPDC7	DEP domain containing 7	-41.39	-1.03
chr11:44332300-44332851	ALX4	ALX homeobox 4	ND	ND
chr11:46368377-46368845	DGKZ	diacylglycerol kinase, zeta	ND	ND
chr11:61062628-61063300	VWCE	von Willebrand factor C and EGF domains	ND	ND
chr11:62369462-62369992	MTA2	metastasis associated 1 family, member 2	-5.77	1.87
chr11:62473956-62475052	GNG3	guanine nucleotide binding protein (G protein), gamma 3	ND	ND
chr11:64878545-64879247	TM7SF2	transmembrane 7 superfamily member 2	-18.22	2.45
chr11:68451192-68451743	GAL	galanin prepropeptide	ND	ND
chr11:75141660-75142510	KLHL35	kelch-like 35 (Drosophila)	ND	ND
chr11:94134588-94135139	GPR83	G protein-coupled receptor 83	ND	ND
chr11:105480696-105481890	GRIA4	glutamate receptor, ionotropic, AMPA 4	ND	ND
chr11:111410865-111411203	LAYN	layilin	ND	ND
chr11:134145496-134145934	GLB1L3	galactosidase, beta 1-like 3	ND	ND
chr12:2903162-2903597	FKBP4	FK506 binding protein 4, 59kDa	-2.17	1.71
chr12:14926217-14928411	H2AFJ	H2A histone family, member J	ND	1.51
chr12:27396610-27397048	STK38L	serine/threonine kinase 38 like	-1.38	1.90
chr12:27485283-27485733	ARNTL2	aryl hydrocarbon receptor nuclear translocator-like 2	-106.57	1.45
chr12:46663347-46663960	SLC38A1	solute carrier family 38, member 1	-90.75	1.57
chr12:48153421-48153639	RAPGEF3	Rap guanine nucleotide exchange factor (GEF) 3	ND	ND
chr12:48298934-48299651	VDR	vitamin D (1,25- dihydroxyvitamin D3) receptor	-21.92	-1.33

chr12:48398520-48398801	COL2A1	collagen, type II, alpha 1	ND	ND
chr12:50354766-50354999	AQP5	aquaporin 5	ND	ND
chr12:51420407-51420960	SLC11A2	solute carrier family 11 (proton-coupled divalent metal ion transporters), member 2	ND	ND
chr12:52626711-52626944	KRT7	keratin 7	ND	ND
chr12:52685180-52685462	KRT81	keratin 81	-14.31	3.84
chr12:54785193-54785432	ZNF385A	zinc finger protein 385A	ND	ND
chr12:65515587-65516303	WIF1	WNT inhibitory factor 1	ND	ND
chr12:75905536-75905790	KRR1	KRR1, small subunit (SSU) processome component, homolog (yeast)	-7.07	3.02
chr12:81471371-81472274	ACSS3	acyl-CoA synthetase short-chain family member 3	ND	ND
chr12:95942736-95943223	USP44	ubiquitin specific peptidase 44	ND	ND
chr12:96184910-96185477	NTN4	netrin 4	-43.85	2.29
chr12:96251547-96252318	SNRPF	small nuclear ribonucleoprotein polypeptide F	-2.94	1.02
chr12:104532428-104532774	NFYB	nuclear transcription factor Y, beta	-8.14	1.04
chr12:105478470-105479342	ALDH1L2	aldehyde dehydrogenase 1 family, member L2	1.22	-1.02
chr12:106533526-106533976	NUAK1	NUAK family, SNF1-like kinase, 1	-24.94	3.96
chr12:121647074-121647718	P2RX4	purinergic receptor P2X, ligand-gated ion channel, 4	1.37	1.01
chr12:123380768-123381235	VPS37B	vacuolar protein sorting 37 homolog B (S. cerevisiae)	-6.85	1.52
chr12:130822320-130822708	PIWIL1	piwi-like 1 (Drosophila)	ND	ND
chr13:29293235-29293583	SLC46A3	solute carrier family 46, member 3	ND	11.85
chr13:44947258-44947609	SERP2	stress-associated endoplasmic reticulum protein family member 2	ND	ND
chr14:21494052-21494384	NDRG2	NDRG family member 2	ND	2.79

chr14:31342847-31343434	COCH	coagulation factor C homolog, cochlin (Limulus polyphemus)	ND	ND
chr14:37052136-37053767	NKX2-8	NK2 homeobox 8	ND	ND
chr14:38723836-38725853	CLEC14A	C-type lectin domain family 14, member A	ND	ND
chr14:50155103-50155452	POLE2	polymerase (DNA directed), epsilon 2, accessory subunit	$-\infty$	-8.28
chr14:52536167-52536798	NID2	nidogen 2 (osteonidogen)	-7.46	-2.00
chr14:59932053-59932367	GPR135	G protein-coupled receptor 135	ND	ND
chr14:69725812-69726434	GALNTL1	UDP-N-acetyl-alpha-D-galactosamine:polypeptide N-acetylgalactosaminyltransferase-like 1	ND	ND
chr14:75422551-75422954	PGF	placental growth factor	-4.99	13.18
chr14:89259436-89259978	EML5	echinoderm microtubule associated protein like 5	ND	ND
chr14:100258701-100259279	EML1	echinoderm microtubule associated protein like 1	-33.25	-4.30
chr14:101192769-101193133	DLK1	delta-like 1 homolog (Drosophila)	ND	ND
chr14:105952509-105953465	CRIP1	cysteine-rich protein 1 (intestinal)	ND	ND
chr15:31195574-31196251	FAN1	FANCD2/FANCI-associated nuclease 1	ND	ND
chr15:33008828-33010062	GREM1	gremlin 1	ND	ND
chr15:37110159-37110906	CSNK1A1P1	casein kinase 1, alpha 1 pseudogene 1	ND	ND
chr15:38857321-38857752	RASGRP1	RAS guanyl releasing protein 1 (calcium and DAG-regulated)	ND	ND
chr15:40545185-40545996	C15orf56	chromosome 15 open reading frame 56	ND	ND
chr15:41135626-41135983	SPINT1	serine peptidase inhibitor, Kunitz type 1	ND	ND
chr15:41951920-41953401	MGA	MAX gene associated	-3.33	-2.16
chr15:45670914-45671287	GATM	glycine amidinotransferase (L-arginine:glycine amidinotransferase)	ND	ND
chr15:45926647-45927017	SQRDL	sulfide quinone reductase-like (yeast)	-41.33	2.27

chr15:50474044-50474269	SLC27A2	solute carrier family 27 (fatty acid transporter), member 2	ND	ND
chr15:52404916-52405519	BCL2L10	BCL2-like 10 (apoptosis facilitator)	ND	ND
chr15:63333735-63334478	TPM1	tropomyosin 1 (alpha)	-183.15	-2.10
chr15:65715564-65715910	IGDCC4	immunoglobulin superfamily, DCC subclass, member 4	ND	ND
chr15:69744146-69745019	RPLP1	ribosomal protein, large, P1	-4.80	1.54
chr15:73661653-73662113	HCN4	hyperpolarization activated cyclic nucleotide-gated potassium channel 4	ND	ND
chr15:74726431-74726832	SEMA7A	semaphorin 7A, GPI membrane anchor (John Milton Hagen blood group)	ND	5.61
chr15:75135934-75136206	ULK3	unc-51-like kinase 3 (C. elegans)	-2.96	1.42
chr15:79104085-79104725	ADAMTS7	ADAM metallopeptidase with thrombospondin type 1 motif, 7	ND	ND
chr15:83953662-83954106	BNC1	basonuclin 1	ND	ND
chr15:90743903-90744283	SEMA4B	sema domain, immunoglobulin domain (Ig), transmembrane domain (TM) and short cytoplasmic domain, (semaphorin) 4B	ND	ND
chr15:91071782-91072672	CRTC3	CREB regulated transcription coactivator 3	-2.81	1.01
chr15:93632661-93633317	RGMA	RGM domain family, member A	ND	ND
chr15:100273911-100274649	LYSMD4	LysM, putative peptidoglycan-binding, domain containing 4	-3.46	1.10
chr15:101084954-101085225	CERS3	ceramide synthase 3	ND	ND
chr15:102030601-102030913	PCSK6	proprotein convertase subtilisin/kexin type 6	ND	ND
chr16:1542348-1543132	TELO2	TEL2, telomere maintenance 2, homolog (S. cerevisiae)	-4.20	1.50
chr16:2284870-2285784	DNASE1L2	deoxyribonuclease I-like 2	ND	ND
chr16:2568947-2570101	AMDHD2	amidohydrolase domain containing 2	-6.92	1.91

chr16:3067958-3069459	CLDN6	claudin 6	ND	ND
chr16:3354783-3354980	ZNF75A	zinc finger protein 75a	-2.62	1.72
chr16:4665096-4666153	FAM100A	family with sequence similarity 100, member A	-1.05	1.26
chr16:4987455-4988023	PPL	periplakin	ND	2.22
chr16:19896439-19897434	GPRC5B	G protein-coupled receptor, family C, group 5, member B	-1.63	-1.58
chr16:29984585-29984869	TAOK2	TAO kinase 2	-4.41	2.41
chr16:30107736-30108138	YPEL3	yippee-like 3 (Drosophila)	-10.61	2.97
chr16:30905688-30906027	BCL7C	B-cell CLL/lymphoma 7C	-3.96	4.08
chr16:31214220-31214691	PYCARD	PYD and CARD domain containing	ND	ND
chr16:55690311-55690741	SLC6A2	solute carrier family 6 (neurotransmitter transporter, noradrenalin), member 2	ND	ND
chr16:56622825-56623153	MT3	metallothionein 3	ND	ND
chr16:56650538-56651661	MT1L	metallothionein 1L (gene/pseudogene)	ND	ND
chr16:56658877-56659551	MT1E	metallothionein 1E	-80.38	1.84
chr16:56665864-56666360	MT1M	metallothionein 1M	ND	ND
chr16:56671745-56672493	MT1A	metallothionein 1A	ND	ND
chr16:56715795-56716107	MT1X	metallothionein 1X	$-\infty$	2.40
chr16:66459884-66460660	BEAN1	brain expressed, associated with NEDD4, 1	ND	ND
chr16:66612405-66613125	CMTM2	CKLF-like MARVEL transmembrane domain containing 2	ND	ND
chr16:66877964-66878238	CA7	carbonic anhydrase VII	ND	ND
chr16:66959243-66959891	RRAD	Ras-related associated with diabetes	ND	232.66
chr16:67142849-67143400	C16orf70	chromosome 16 open reading frame 70	-4.63	1.37
chr16:67427844-67428472	TPPP3	tubulin polymerization-promoting protein family member 3	ND	ND

chr16:68002913-68003264	SLC12A4	solute carrier family 12 (potassium/chloride transporters), member 4	-7.56	1.11
chr16:68269942-68271096	ESRP2	epithelial splicing regulatory protein 2	ND	ND
chr16:75019257-75019801	WDR59	WD repeat domain 59	-5.39	1.53
chr16:83841075-83841393	HSBP1	heat shock factor binding protein 1	-8.85	1.41
chr16:84852183-84853379	CRISPLD2	cysteine-rich secretory protein LCCL domain containing 2	ND	5.19
chr16:89159482-89160090	ACSF3	acyl-CoA synthetase family member 3	ND	ND
chr17:1932853-1933310	DPH1	DPH1 homolog (S. cerevisiae)	-5.36	1.02
chr17:3796786-3797058	CAMKK1	calcium/calmodulin-dependent protein kinase kinase 1, alpha	ND	ND
chr17:4981292-4981732	ZFP3	zinc finger protein 3 homolog (mouse)	ND	ND
chr17:6898486-6899373	ALOX12	arachidonate 12-lipoxygenase	ND	ND
chr17:6947248-6947774	SLC16A11	solute carrier family 16, member 11 (monocarboxylic acid transporter 11)	ND	ND
chr17:7832860-7833744	KCNAB3	potassium voltage-gated channel, shaker-related subfamily, beta member 3	ND	ND
chr17:14206611-14207430	MGC12916	uncharacterized protein MGC12916	ND	ND
chr17:16592773-16593334	CCDC144A	coiled-coil domain containing 144A	ND	ND
chr17:19648383-19649285	ALDH3A1	aldehyde dehydrogenase 3 family, member A1	ND	ND
chr17:19771313-19772288	ULK2	unc-51-like kinase 2 (C. elegans)	-1.99	-2.01
chr17:20058481-20058999	SPECC1	sperm antigen with calponin homology and coiled-coil domains 1	ND	3.41
chr17:26634229-26634548	KRT18P55	keratin 18 pseudogene 55	ND	ND
chr17:28705112-28705501	CPD	carboxypeptidase D	-10.99	-1.00
chr17:33775426-33776966	SLFN13	schlafen family member 13	ND	ND
chr17:34947743-34948007	DHRS11	dehydrogenase/reductase (SDR family) member 11	-9.04	1.96

chr17:36105367-36105779	HNF1B	HNF1 homeobox B	ND	ND
chr17:38083994-38084555	ORMDL3	ORM1-like 3 (S. cerevisiae)	-1.76	2.46
chr17:38211029-38211245	MED24	mediator complex subunit 24	-3.19	1.26
chr17:39684037-39685016	KRT19	keratin 19	ND	ND
chr17:40687730-40687991	NAGLU	N-acetylglucosaminidase, alpha	-1.24	1.06
chr17:40932147-40932502	WNK4	WNK lysine deficient protein kinase 4	-84.19	-2.21
chr17:42835670-42836026	ADAM11	ADAM metallopeptidase domain 11	ND	ND
chr17:43046147-43046525	C1QL1	complement component 1, q subcomponent-like 1	-97.74	-1.52
chr17:43298239-43298900	FMNL1	formin-like 1	ND	ND
chr17:44896356-44897128	WNT3	wingless-type MMTV integration site family, member 3	ND	ND
chr17:44928191-44928746	WNT9B	wingless-type MMTV integration site family, member 9B	ND	ND
chr17:47439942-47440319	ZNF652	zinc finger protein 652	-2.93	-1.78
chr17:48172012-48172477	PDK2	pyruvate dehydrogenase kinase, isozyme 2	-1.72	-1.28
chr17:48585158-48585555	MYCBPAP	MYCBP associated protein	ND	ND
chr17:56565851-56566106	HSF5	heat shock transcription factor family member 5	ND	ND
chr17:61553311-61553848	ACE	angiotensin I converting enzyme (peptidyl-dipeptidase A) 1	ND	ND
chr17:67323724-67324044	ABCA5	ATP-binding cassette, sub-family A (ABC1), member 5	-3.29	-1.15
chr17:72269849-72270350	DNAI2	dynein, axonemal, intermediate chain 2	ND	ND
chr17:73893284-73893696	TRIM65	tripartite motif containing 65	-5.67	1.72
chr17:74863820-74864272	MGAT5B	mannosyl (alpha-1,6-)-glycoprotein beta-1,6-N-acetyl-glucosaminyltransferase, isozyme B	ND	ND
chr17:77813784-77814385	CBX4	chromobox homolog 4	-4.75	2.09

chr17:79869635-79870860	PCYT2	phosphate cytidylyltransferase 2, ethanolamine	-11.83	1.69
chr18:9707367-9708058	RAB31	RAB31, member RAS oncogene family	-4.85	1.40
chr18:10453576-10454261	APCDD1	adenomatosis polyposis coli down-regulated 1	ND	ND
chr18:12307146-12307787	TUBB6	tubulin, beta 6 class V	-11.86	2.10
chr18:22006073-22006563	IMPACT	Impact homolog (mouse)	-2.96	1.67
chr18:28622998-28623283	DSC3	desmocollin 3	ND	ND
chr18:47086920-47088880	LIPG	lipase, endothelial	ND	ND
chr18:56941107-56941501	RAX	retina and anterior neural fold homeobox	ND	ND
chr18:59991707-59992259	TNFRSF11A	tumor necrosis factor receptor superfamily, member 11a, NFKB activator	-46.36	-2.05
chr18:74535345-74536050	ZNF236	zinc finger protein 236	-4.88	-1.45
chr18:74961717-74962155	GALR1	galanin receptor 1	ND	ND
chr18:76828285-76828813	ATP9B	ATPase, class II, type 9B	-2.50	1.23
chr18:77623089-77623556	KCNG2	potassium voltage-gated channel, subfamily G, member 2	ND	ND
chr18:77723553-77724356	HSBP1L1	heat shock factor binding protein 1-like 1	-16.96	1.40
chr19:375772-376277	THEG	theg spermatid protein	ND	ND
chr19:1238317-1239966	C19orf26	chromosome 19 open reading frame 26	ND	ND
chr19:1268003-1268914	CIRBP	cold inducible RNA binding protein	-2.13	1.48
chr19:1523488-1523868	PLK5	polo-like kinase 5	ND	ND
chr19:2702871-2703194	GNG7	guanine nucleotide binding protein (G protein), gamma 7	-1.61	-2.48
chr19:2721607-2722400	DIRAS1	DIRAS family, GTP-binding RAS-like 1	-2.88	-1.06
chr19:3178212-3178758	S1PR4	sphingosine-1-phosphate receptor 4	ND	ND
chr19:4908726-4909291	UHRF1	ubiquitin-like with PHD and ring finger domains 1	$-\infty$	-4.78

chr19:6767934-6768318	SH2D3A	SH2 domain containing 3A	ND	ND
chr19:10614089-10614570	KEAP1	kelch-like ECH-associated protein 1	-5.02	2.06
chr19:10947290-10947723	TMED1	transmembrane emp24 protein transport domain containing 1	-9.72	2.93
chr19:18699104-18699447	C19orf60	chromosome 19 open reading frame 60	-3.38	1.64
chr19:19281287-19281758	MEF2B	myocyte enhancer factor 2B	ND	ND
chr19:19639225-19639701	YJEFN3	YjeF N-terminal domain containing 3	ND	ND
chr19:19648063-19648617	CILP2	cartilage intermediate layer protein 2	ND	ND
chr19:35633146-35633671	FXYD7	FXYD domain containing ion transport regulator 7	ND	ND
chr19:36523759-36524120	CLIP3	CAP-GLY domain containing linker protein 3	ND	ND
chr19:37824989-37825523	HKR1	HKR1, GLI-Kruppel zinc finger family member	ND	ND
chr19:38810000-38810404	KCNK6	potassium channel, subfamily K, member 6	ND	12.35
chr19:39522838-39523634	FBXO27	F-box protein 27	ND	ND
chr19:40324237-40325513	DYRK1B	dual-specificity tyrosine-(Y)-phosphorylation regulated kinase 1B	ND	ND
chr19:42348209-42349263	LYPD4	LY6/PLAUR domain containing 4	ND	ND
chr19:45907294-45908668	PPP1R13L	protein phosphatase 1, regulatory subunit 13 like	ND	ND
chr19:46318682-46319297	RSPH6A	radial spoke head 6 homolog A (Chlamydomonas)	ND	ND
chr19:46799961-46800276	HIF3A	hypoxia inducible factor 3, alpha subunit	ND	ND
chr19:49116353-49117024	FAM83E	family with sequence similarity 83, member E	ND	ND
chr19:49340366-49340896	HSD17B14	hydroxysteroid (17-beta) dehydrogenase 14	16.06	ND
chr19:49828421-49828900	SLC6A16	solute carrier family 6, member 16	ND	ND

chr19:49891206-49891808	CCDC155	coiled-coil domain containing 155	ND	ND
chr19:50978885-50979420	EMC10	ER membrane protein complex subunit 10	1.04	3.29
chr19:55791875-55792127	HSPBP1	HSPA (heat shock 70kDa) binding protein, cytoplasmic cochaperone 1	ND	4.78
chr19:57741797-57742575	AURKC	aurora kinase C	ND	ND
chr19:58094925-58095320	ZIK1	zinc finger protein interacting with K protein 1 homolog (mouse)	ND	ND
chr19:58629862-58630290	ZSCAN18	zinc finger and SCAN domain containing 18	ND	ND
chr19:58951813-58952603	ZNF132	zinc finger protein 132	ND	ND
chr2:3642071-3642741	COLEC11	collectin sub-family member 11	ND	ND
chr2:9613758-9614409	IAH1	isoamyl acetate-hydrolyzing esterase 1 homolog (S. cerevisiae)	-7.20	1.09
chr2:10588933-10589544	ODC1	ornithine decarboxylase 1	-8.99	2.80
chr2:11886034-11886510	LPIN1	lipin 1	-6.19	-1.46
chr2:25475594-25476217	DNMT3A	DNA (cytosine-5-)-methyltransferase 3 alpha	ND	ND
chr2:27603642-27604352	ZNF513	zinc finger protein 513	-2.52	3.85
chr2:37571597-37571868	QPCT	glutaminyl-peptide cyclotransferase	5.18	3.72
chr2:38302404-38303508	CYP1B1	cytochrome P450, family 1, subfamily B, polypeptide 1	-22.71	2.67
chr2:39187695-39188062	LOC375196	uncharacterized LOC375196	ND	ND
chr2:42395594-42396266	EML4	echinoderm microtubule associated protein like 4	-15.46	1.12
chr2:43019952-43020328	HAAO	3-hydroxyanthranilate 3,4-dioxygenase	ND	ND
chr2:54087223-54087627	GPR75-ASB3	GPR75-ASB3 readthrough	-6.24	4.34
chr2:56150181-56150620	EFEMP1	EGF containing fibulin-like extracellular matrix protein 1	-253.30	-1.67
chr2:71680378-71681062	DYSF	dysferlin, limb girdle muscular dystrophy 2B (autosomal recessive)	ND	ND

chr2:74669154-74669651	RTKN	rhotekin	ND	3.79
chr2:74740921-74741473	TLX2	T-cell leukemia homeobox 2	ND	ND
chr2:75938201-75938442	GCFC2	GC-rich sequence DNA-binding factor 2	-5.64	-1.33
chr2:85979886-85980833	ATOH8	atonal homolog 8 (Drosophila)	-42.81	-2.25
chr2:106015808-106016964	FHL2	four and a half LIM domains 2	ND	9.75
chr2:111489528-111490228	ACOXL	acyl-CoA oxidase-like	ND	ND
chr2:113992626-113993488	LOC654433	uncharacterized LOC654433	ND	25.32
chr2:119605987-119606276	EN1	engrailed homeobox 1	ND	ND
chr2:124782154-124782642	CNTNAP5	contactin associated protein-like 5	ND	ND
chr2:136743371-136743706	DARS	aspartyl-tRNA synthetase	-7.72	1.29
chr2:160761331-160761787	LY75	lymphocyte antigen 75	ND	ND
chr2:160919067-160919700	PLA2R1	phospholipase A2 receptor 1, 180kDa	ND	ND
chr2:176956305-176957219	HOXD13	homeobox D13	-∞	1.99
chr2:176963873-176964672	HOXD12	homeobox D12	ND	ND
chr2:176980582-176981411	HOXD10	homeobox D10	ND	ND
chr2:183731724-183732269	FRZB	frizzled-related protein	ND	ND
chr2:190445949-190446453	SLC40A1	solute carrier family 40 (iron-regulated transporter), member 1	ND	ND
chr2:191044843-191045016	C2orf88	chromosome 2 open reading frame 88	ND	ND
chr2:198651005-198651479	BOLL	bol, boule-like (Drosophila)	ND	ND
chr2:201450427-201450786	AOX1	aldehyde oxidase 1	-16.40	2.64
chr2:206546253-206546632	NRP2	neuropilin 2	ND	ND
chr2:219723559-219724119	WNT6	wingless-type MMTV integration site family, member 6	ND	ND
chr2:220083902-220084432	ABCB6	ATP-binding cassette, sub-family B (MDR/TAP), member 6	-8.93	1.05
chr2:220117392-220118010	TUBA4B	tubulin, alpha 4b (pseudogene)	ND	ND
chr2:223288457-223288946	SGPP2	sphingosine-1-phosphate phosphatase 2	ND	ND
chr2:232395277-232395800	NMUR1	neuromedin U receptor 1	ND	ND
chr2:232791537-232792428	NPPC	natriuretic peptide C	ND	8.26

chr2:233352703-233353049	ECEL1	endothelin converting enzyme-like 1	ND	ND
chr2:236401725-236402264	AGAP1	ArfGAP with GTPase domain, ankyrin repeat and PH domain 1	-23.73	-1.48
chr2:237416359-237416798	IQCA1	IQ motif containing with AAA domain 1	ND	ND
chr2:238706857-238707407	RBM44	RNA binding motif protein 44	ND	ND
chr2:238767598-238768051	RAMP1	receptor (G protein-coupled) activity modifying protein 1	-10.31	2.80
chr2:241497475-241498016	ANKMY1	ankyrin repeat and MYND domain containing 1	ND	ND
chr20:3051998-3052439	OXT	oxytocin, prepropeptide	ND	ND
chr20:3662928-3663345	ADAM33	ADAM metallopeptidase domain 33	ND	ND
chr20:22566497-22567156	FOXA2	forkhead box A2	ND	ND
chr20:23015394-23015797	SSTR4	somatostatin receptor 4	ND	ND
chr20:30639366-30640247	HCK	hemopoietic cell kinase	ND	ND
chr20:34359412-34359810	PHF20	PHD finger protein 20	-5.85	1.12
chr20:35201122-35201709	TGIF2	TGFB-induced factor homeobox 2	-9.89	-2.46
chr20:35579740-35580525	SAMHD1	SAM domain and HD domain 1	-4.31	1.96
chr20:36148527-36149309	NNAT	neuronatin	ND	ND
chr20:40247721-40248041	CHD6	chromodomain helicase DNA binding protein 6	-4.63	-2.23
chr20:42544470-42544897	TOX2	TOX high mobility group box family member 2	ND	ND
chr20:43729497-43729969	KCNS1	potassium voltage-gated channel, delayed-rectifier, subfamily S, member 1	ND	ND
chr20:44098004-44098710	WFDC2	WAP four-disulfide core domain 2	ND	ND
chr20:48770695-48770956	TMEM189	transmembrane protein 189	-13.74	1.64
chr20:55904407-55905220	SPO11	SPO11 meiotic protein covalently bound to DSB homolog (S. cerevisiae)	ND	ND
chr20:57266936-57267562	NPEPL1	aminopeptidase-like 1	ND	ND
chr20:61558154-61558557	DIDO1	death inducer-obliterator 1	-14.71	1.13

chr20:61886125-61886472	NKAIN4	Na+/K+ transporting ATPase interacting 4	ND	ND
chr20:61993075-61993388	CHRNA4	cholinergic receptor, nicotinic, alpha 4 (neuronal)	ND	ND
chr20:62179216-62179889	SRMS	src-related kinase lacking C-terminal regulatory tyrosine and N-terminal myristylation sites	ND	ND
chr20:62611097-62611420	SAMD10	sterile alpha motif domain containing 10	ND	ND
chr20:62611717-62612236	PRPF6	PRP6 pre-mRNA processing factor 6 homolog (S. cerevisiae)	-4.38	2.12
chr21:10991122-10991595	TPTE	transmembrane phosphatase with tensin homology	ND	ND
chr21:18985560-18985907	BTG3	BTG family, member 3	-1.54	1.65
chr21:30670144-30670803	BACH1	BTB and CNC homology 1, basic leucine zipper transcription factor 1	-17.08	-1.08
chr21:35747246-35747584	FAM165B	family with sequence similarity 165, member B	-4.47	1.72
chr21:35832055-35832925	KCNE1	potassium voltage-gated channel, Isk-related family, member 1	ND	ND
chr21:38377682-38378685	DSCR6	Down syndrome critical region gene 6	ND	ND
chr21:43917041-43917360	RSPH1	radial spoke head 1 homolog (Chlamydomonas)	ND	ND
chr21:45137804-45138387	PDXK	pyridoxal (pyridoxine, vitamin B6) kinase	-9.71	1.31
chr21:46823754-46824561	COL18A1	collagen, type XVIII, alpha 1	-20.35	-2.37
chr21:46962888-46963536	SLC19A1	solute carrier family 19 (folate transporter), member 1	-4.59	-1.76
chr22:17602363-17602826	CECR6	cat eye syndrome chromosome region, candidate 6	ND	ND

chr22:19709696-19710753	GP1BB	glycoprotein Ib (platelet), beta polypeptide	ND	ND
chr22:19743166-19744165	TBX1	T-box 1	ND	ND
chr22:28198084-28198677	MN1	meningioma (disrupted in balanced translocation) 1	ND	ND
chr22:29601418-29601637	EMID1	EMI domain containing 1	ND	ND
chr22:29711800-29712550	RASL10A	RAS-like, family 10, member A	ND	ND
chr22:29875828-29875980	NEFH	neurofilament, heavy polypeptide	ND	ND
chr22:31064169-31064718	DUSP18	dual specificity phosphatase 18	ND	3.03
chr22:39852086-39852947	MGAT3	mannosyl (beta-1,4-)-glycoprotein beta-1,4-N-acetylglucosaminyltransferase	ND	ND
chr22:40390353-40390683	FAM83F	family with sequence similarity 83, member F	ND	ND
chr22:42195654-42196128	CCDC134	coiled-coil domain containing 134	-7.26	1.03
chr22:43045713-43046044	CYB5R3	cytochrome b5 reductase 3	-1.77	1.09
chr22:43546923-43547376	TSPO	translocator protein (18kDa)	-6.16	1.64
chr22:44287984-44288413	PNPLA5	patatin-like phospholipase domain containing 5	ND	ND
chr22:45704820-45705468	FAM118A	family with sequence similarity 118, member A	ND	ND
chr22:45808748-45810120	SMC1B	structural maintenance of chromosomes 1B	ND	ND
chr22:46067032-46067418	ATXN10	ataxin 10	-8.14	1.53
chr3:15374237-15374679	SH3BP5	SH3-domain binding protein 5 (BTK-associated)	-1.10	2.89
chr3:23243827-23244233	UBE2E2	ubiquitin-conjugating enzyme E2E 2	-3.31	1.16
chr3:40350529-40351025	EIF1B	eukaryotic translation initiation factor 1B	-1.75	1.17
chr3:42543310-42543995	VIPR1	vasoactive intestinal peptide receptor 1	-34.03	-2.37
chr3:52278653-52279601	PPM1M	protein phosphatase, Mg2+/Mn2+ dependent, 1M	-6.70	1.56

chr3:94656579-94656910	LOC255025	uncharacterized LOC255025	ND	ND
chr3:94656940-94657250	LOC255025	uncharacterized LOC255025	ND	ND
chr3:107810307-107810954	CD47	CD47 molecule	-2.95	1.40
chr3:108836657-108837192	MORC1	MORC family CW-type zinc finger 1	ND	ND
chr3:112051827-112052643	CD200	CD200 molecule	-178.00	-2.04
chr3:112709147-112709631	GTPBP8	GTP-binding protein 8 (putative)	-20.63	1.71
chr3:120170171-120170626	FSTL1	follistatin-like 1	-2.59	1.35
chr3:128212030-128212678	GATA2	GATA binding protein 2	ND	ND
chr3:128713063-128713224	KIAA1257	KIAA1257	ND	ND
chr3:136537464-136537916	SLC35G2	solute carrier family 35, member G2	ND	5.92
chr3:138665666-138666813	C3orf72	chromosome 3 open reading frame 72	-21.16	1.26
chr3:139258200-139259223	RBP1	retinol binding protein 1, cellular	ND	ND
chr3:139653361-139653871	CLSTN2	calsyntenin 2	ND	ND
chr3:140949673-140950188	ACPL2	acid phosphatase-like 2	-50.21	1.08
chr3:145879251-145879830	PLOD2	procollagen-lysine, 2-oxoglutarate 5-dioxygenase 2	-13.07	1.49
chr3:148804534-148805355	HLTF	helicase-like transcription factor	-8.22	-2.10
chr3:155572573-155573697	SLC33A1	solute carrier family 33 (acetyl-CoA transporter), member 1	-4.53	1.03
chr3:158450446-158450918	RARRES1	retinoic acid receptor responder (tazarotene induced) 1	ND	ND
chr3:160167122-160168425	TRIM59	tripartite motif containing 59	ND	ND
chr3:170135579-170136607	CLDN11	claudin 11	ND	ND
chr3:170626510-170627261	EIF5A2	eukaryotic translation initiation factor 5A2	-1.55	2.67
chr3:197025725-197026507	DLG1	discs, large homolog 1 (Drosophila)	-4.12	1.10
chr4:683006-683622	MFSD7	major facilitator superfamily domain containing 7	ND	ND
chr4:8270376-8271310	HTRA3	HtrA serine peptidase 3	ND	ND
chr4:11430715-11431624	HS3ST1	heparan sulfate (glucosamine) 3-O-sulfotransferase 1	ND	ND

chr4:15657588-15657928	FBXL5	F-box and leucine-rich repeat protein 5	-2.60	1.46
chr4:16085488-16085930	PROM1	prominin 1	ND	ND
chr4:39368264-39368762	RFC1	replication factor C (activator 1) 1, 145kDa	-10.51	1.14
chr4:40632097-40632959	RBM47	RNA binding motif protein 47	ND	ND
chr4:41258379-41259212	UCHL1	ubiquitin carboxyl-terminal esterase L1 (ubiquitin thiolesterase)	54.74	ND
chr4:48908628-48909155	OCIAD2	OCIA domain containing 2	ND	ND
chr4:53727368-53727997	RASL11B	RAS-like, family 11, member B	ND	ND
chr4:55523022-55523948	KIT	v-kit Hardy-Zuckerman 4 feline sarcoma viral oncogene homolog	84.64	ND
chr4:57522307-57523104	HOPX	HOP homeobox	ND	ND
chr4:79472427-79472826	ANXA3	annexin A3	-36.28	3.21
chr4:81117918-81118400	PRDM8	PR domain containing 8	ND	ND
chr4:110480679-110480888	CCDC109B	coiled-coil domain containing 109B	-12.10	1.88
chr4:111544276-111544744	PITX2	paired-like homeodomain 2	ND	ND
chr4:142557239-142557527	IL15	interleukin 15	-18.16	-1.02
chr4:145565950-145566746	HHIP	hedgehog interacting protein	-17.47	-1.74
chr4:154604859-154605399	TLR2	toll-like receptor 2	ND	ND
chr4:155702242-155702610	RBM46	RNA binding motif protein 46	ND	ND
chr4:170192677-170193182	SH3RF1	SH3 domain containing ring finger 1	-8.50	1.46
chr4:190860777-190861654	FRG1	FSHD region gene 1	-5.44	1.73
chr5:1295445-1296578	TERT	telomerase reverse transcriptase	ND	ND
chr5:1800198-1800769	MRPL36	mitochondrial ribosomal protein L36	-4.84	2.46
chr5:9546475-9547064	SEMA5A	sema domain, seven thrombospondin repeats (type 1 and type 1-like), transmembrane domain (TM) and short cytoplasmic domain, (semaphorin) 5A	1.03	1.53
chr5:10761788-10762243	DAP	death-associated protein	-9.26	2.14
chr5:34838853-34839172	TTC23L	tetratricopeptide repeat domain 23-like	ND	ND
chr5:37834728-37835589	GDNF	glial cell derived neurotrophic factor	-51.04	-1.23

chr5:41870875-41871264	OXCT1	3-oxoacid CoA transferase 1	-1.34	2.05
chr5:67584080-67584324	PIK3R1	phosphoinositide-3-kinase, regulatory subunit 1 (alpha)	ND	ND
chr5:72415351-72415999	TMEM171	transmembrane protein 171	-24.87	1.07
chr5:73936842-73938231	ENC1	ectodermal-neural cortex 1 (with BTB-like domain)	-2.00	4.08
chr5:76934706-76935354	OTP	orthopedia homeobox	ND	ND
chr5:87980826-87981595	LINC00461	long intergenic non-protein coding RNA 461	ND	ND
chr5:95768686-95769179	PCSK1	proprotein convertase subtilisin/kexin type 1	ND	ND
chr5:121187285-121187780	FTMT	ferritin mitochondrial	ND	ND
chr5:122110299-122110486	SNX2	sorting nexin 2	-2.99	1.70
chr5:128794982-128795775	ADAMTS19	ADAM metallopeptidase with thrombospondin type 1 motif, 19	ND	ND
chr5:131892031-131892535	RAD50	RAD50 homolog (S. cerevisiae)	-15.36	-1.12
chr5:132113406-132114002	SEPT8	septin 8	-9.14	-1.45
chr5:134735273-134736228	H2AFY	H2A histone family, member Y	-7.77	1.21
chr5:140734634-140735117	PCDHGA4	protocadherin gamma subfamily A, 4	ND	ND
chr5:140787363-140787749	PCDHGB6	protocadherin gamma subfamily B, 6	ND	ND
chr5:149545797-149546251	CDX1	caudal type homeobox 1	ND	ND
chr5:151138457-151138842	ATOX1	ATX1 antioxidant protein 1 homolog (yeast)	-6.73	2.71
chr5:156886889-156887195	NIPAL4	NIPA-like domain containing 4	ND	ND
chr5:159342764-159343255	ADRA1B	adrenoceptor alpha 1B	ND	3.06
chr5:159797723-159798014	C1QTNF2	C1q and tumor necrosis factor related protein 2	ND	ND
chr5:174870496-174871687	DRD1	dopamine receptor D1	ND	ND
chr5:178986372-178987379	RUFY1	RUN and FYVE domain containing 1	ND	1.70
chr5:180632569-180633525	TRIM7	tripartite motif containing 7	ND	3.80
chr6:1388911-1389627	FOXF2	forkhead box F2	-5.90	1.03

chr6:2903839-2904044	SERPINB9	serpin peptidase inhibitor, clade B (ovalbumin), member 9	-14.03	-1.02
chr6:8436172-8436618	SLC35B3	solute carrier family 35, member B3	ND	3.91
chr6:11044847-11045627	ELOVL2	ELOVL fatty acid elongase 2	-15.24	1.02
chr6:16237684-16238572	GMPR	guanosine monophosphate reductase	19.88	4.97
chr6:17600110-17600345	FAM8A1	family with sequence similarity 8, member A1	-3.49	1.52
chr6:19836543-19837425	ID4	inhibitor of DNA binding 4, dominant negative helix-loop-helix protein	-46.13	1.40
chr6:21664380-21667024	LINC00340	long intergenic non-protein coding RNA 340	-7.40	-1.36
chr6:26021689-26022133	HIST1H4A	histone cluster 1, H4a	-46.72	-2.80
chr6:26044123-26045078	HIST1H2BB	histone cluster 1, H2bb	-∞	-2.58
chr6:26188431-26189502	HIST1H4D	histone cluster 1, H4d	-10.86	-3.92
chr6:26272400-26272913	HIST1H2BI	histone cluster 1, H2bi	-44.53	-2.51
chr6:27791300-27791894	HIST1H4J	histone cluster 1, H4j	ND	ND
chr6:30875141-30875574	GTF2H4	general transcription factor IIH, polypeptide 4, 52kDa	ND	ND
chr6:30881453-30881994	VARS2	valyl-tRNA synthetase 2, mitochondrial (putative)	ND	ND
chr6:31465269-31466068	MICB	MHC class I polypeptide-related sequence B	ND	ND
chr6:34203358-34204454	HMGA1	high mobility group AT-hook 1	-32.79	1.51
chr6:36355727-36356281	ETV7	ets variant 7	ND	ND
chr6:41605063-41605669	MDFI	MyoD family inhibitor	-124.54	1.74
chr6:42927543-42928709	GNMT	glycine N-methyltransferase	ND	ND
chr6:43337565-43337887	ZNF318	zinc finger protein 318	-6.45	-1.08
chr6:46097028-46098330	ENPP4	ectonucleotide pyrophosphatase/phosphodiesterase 4 (putative)	ND	ND

chr6:46703038-46703609	PLA2G7	phospholipase A2, group VII (platelet-activating factor acetylhydrolase, plasma)	-59.04	4.04
chr6:54710962-54711524	FAM83B	family with sequence similarity 83, member B	ND	ND
chr6:72130688-72131121	LINC00472	long intergenic non-protein coding RNA 472	-7.26	1.16
chr6:73330570-73331615	KCNQ5	potassium voltage-gated channel, KQT-like subfamily, member 5	ND	ND
chr6:84419138-84419727	SNAP91	synaptosomal-associated protein, 91kDa homolog (mouse)	ND	ND
chr6:86158888-86159511	NT5E	5'-nucleotidase, ecto (CD73)	-38.64	1.19
chr6:94129477-94129788	EPHA7	EPH receptor A7	ND	ND
chr6:97285597-97286009	GPR63	G protein-coupled receptor 63	ND	ND
chr6:100911430-100912219	SIM1	single-minded homolog 1 (Drosophila)	ND	ND
chr6:132722933-132723403	MOXD1	monooxygenase, DBH-like 1	-55.11	1.53
chr6:137366040-137366611	IL20RA	interleukin 20 receptor, alpha	ND	ND
chr6:144329595-144330248	HYMAI	hydatidiform mole associated and imprinted (non-protein coding)	ND	ND
chr6:144470581-144471190	STX11	syntaxin 11	ND	ND
chr6:146864104-146864507	RAB32	RAB32, member RAS oncogene family	2.50	1.62
chr6:150390477-150390827	ULBP3	UL16 binding protein 3	-22.15	1.36
chr6:158243467-158243844	SNX9	sorting nexin 9	-4.04	2.02
chr6:160220819-160221142	PNLDC1	poly(A)-specific ribonuclease (PARN)-like domain containing 1	ND	16.00
chr6:166581888-166582618	T	T, brachyury homolog (mouse)	ND	ND
chr6:168197661-168198197	C6orf123	chromosome 6 open reading frame 123	ND	ND
chr7:914802-915719	GET4	golgi to ER traffic protein 4 homolog (S. cerevisiae)	-11.89	5.50
chr7:6629076-6629479	C7orf26	chromosome 7 open reading frame 26	-8.59	1.05
chr7:12609821-12611451	SCIN	scinderin	17.50	ND

chr7:12725967-12726197	ARL4A	ADP-ribosylation factor-like 4A	-9.03	1.82
chr7:27213747-27214940	HOXA10	homeobox A10	ND	2.99
chr7:28997991-28998720	TRIL	TLR4 interactor with leucine-rich repeats	ND	ND
chr7:30810469-30811028	FAM188B	family with sequence similarity 188, member B	-12.76	1.40
chr7:43797659-43797933	BLVRA	biliverdin reductase A	-7.38	1.23
chr7:55085841-55086380	EGFR	epidermal growth factor receptor	ND	ND
chr7:72741684-72742453	TRIM50	tripartite motif containing 50	ND	ND
chr7:87105268-87105738	ABCB4	ATP-binding cassette, sub-family B (MDR/TAP), member 4	-26.13	-3.83
chr7:89840254-89840814	STEAP2	STEAP family member 2, metalloreductase	ND	ND
chr7:99775406-99776684	STAG3	stromal antigen 3	-28.90	-1.00
chr7:101005455-101006098	EMID2	EMI domain containing 2	ND	ND
chr7:107643981-107644691	LAMB1	laminin, beta 1	-78.29	-1.60
chr7:117067631-117068263	ASZ1	ankyrin repeat, SAM and basic leucine zipper domain containing 1	ND	ND
chr7:120968703-120970006	WNT16	wingless-type MMTV integration site family, member 16	ND	ND
chr7:127225788-127226051	GCC1	GRIP and coiled-coil domain containing 1	-2.94	2.75
chr7:127227681-127228208	ARF5	ADP-ribosylation factor 5	-6.53	2.52
chr7:127671412-127671663	LRRC4	leucine rich repeat containing 4	-3.09	-2.90
chr7:130125522-130126963	MEST	mesoderm specific transcript homolog (mouse)	-24.61	2.24
chr7:137531593-137531965	DGKI	diacylglycerol kinase, iota	ND	ND
chr7:137531967-137532762	DGKI	diacylglycerol kinase, iota	ND	ND
chr7:142984777-142985174	CASP2	caspase 2, apoptosis-related cysteine peptidase	-5.11	1.08
chr7:150019870-150020481	LRRC61	leucine rich repeat containing 61	ND	ND

chr7:151136846-151137601	CRYGN	crystallin, gamma N	ND	ND
chr7:158937954-158938224	VIPR2	vasoactive intestinal peptide receptor 2	ND	ND
chr8:16859402-16860252	FGF20	fibroblast growth factor 20	ND	ND
chr8:18871493-18872325	PSD3	pleckstrin and Sec7 domain containing 3	-10.89	-1.32
chr8:19459581-19460434	CSGALNACT1	chondroitin sulfate N-acetylgalactosaminyltransferase 1	ND	ND
chr8:21881701-21883178	NPM2	nucleophosmin/nucleoplasmin 2	ND	ND
chr8:22132289-22133583	PIWIL2	piwi-like 2 (Drosophila)	ND	ND
chr8:22297664-22298103	PPP3CC	protein phosphatase 3, catalytic subunit, gamma isozyme	-2.40	1.20
chr8:23540595-23540992	NKX3-1	NK3 homeobox 1	ND	7.10
chr8:24771760-24772392	NEFM	neurofilament, medium polypeptide	ND	ND
chr8:33330830-33331126	FUT10	fucosyltransferase 10 (alpha (1,3) fucosyltransferase)	-6.08	-2.03
chr8:38964730-38965771	ADAM32	ADAM metallopeptidase domain 32	ND	ND
chr8:40755062-40755486	ZMAT4	zinc finger, matrin-type 4	ND	ND
chr8:49647095-49648594	EFCAB1	EF-hand calcium binding domain 1	ND	ND
chr8:55046996-55047676	MRPL15	mitochondrial ribosomal protein L15	-3.14	1.00
chr8:56791547-56792263	LYN	v-yes-1 Yamaguchi sarcoma viral related oncogene homolog	-5.14	-1.29
chr8:65711511-65711822	CYP7B1	cytochrome P450, family 7, subfamily B, polypeptide 1	ND	ND
chr8:67344408-67345127	ADHFE1	alcohol dehydrogenase, iron containing, 1	ND	1.47
chr8:71316506-71317120	NCOA2	nuclear receptor coactivator 2	-2.49	1.01
chr8:82191761-82192643	FABP5	fatty acid binding protein 5 (psoriasis-associated)	ND	ND
chr8:82633708-82634210	ZFAND1	zinc finger, AN1-type domain 1	-4.14	1.30
chr8:85096490-85096967	RALYL	RALY RNA binding protein-like	ND	ND
chr8:87081524-87082279	PSKH2	protein serine kinase H2	ND	ND

chr8:91803149-91804006	NECAB1	N-terminal EF-hand calcium binding protein 1	-2.49	-1.06
chr8:94712011-94712327	FAM92A1	family with sequence similarity 92, member A1	-5.13	-1.30
chr8:95961753-95962464	TP53INP1	tumor protein p53 inducible nuclear protein 1	-1.28	3.17
chr8:96145188-96145710	PLEKHF2	pleckstrin homology domain containing, family F (with FYVE domain) member 2	-1.41	1.34
chr8:97656771-97657290	CPQ	carboxypeptidase Q	1.36	1.61
chr8:99076426-99076956	C8orf47	chromosome 8 open reading frame 47	ND	ND
chr8:101118286-101118965	RGS22	regulator of G-protein signaling 22	ND	ND
chr8:102504167-102505110	GRHL2	grainyhead-like 2 (Drosophila)	ND	ND
chr8:104153428-104153945	C8orf56	chromosome 8 open reading frame 56	ND	ND
chr8:117950195-117950681	AARD	alanine and arginine rich domain containing protein	ND	1.81
chr8:119964424-119965130	TNFRSF11B	tumor necrosis factor receptor superfamily, member 11b	-27.90	3.01
chr8:120219890-120220490	MAL2	mal, T-cell differentiation protein 2 (gene/pseudogene)	ND	9.91
chr8:121824727-121825076	SNTB1	syntrophin, beta 1 (dystrophin-associated protein A1, 59kDa, basic component 1)	-21.18	-1.77
chr8:125741072-125741523	MTSS1	metastasis suppressor 1	1.62	ND
chr8:128805964-128806425	PVT1	Pvt1 oncogene (non-protein coding)	-3.38	1.28
chr8:139509640-139510253	FAM135B	family with sequence similarity 135, member B	ND	ND
chr8:143915521-143916391	GML	glycosylphosphatidylinositol anchored molecule like protein	ND	ND
chr8:144789907-144790748	CCDC166	coiled-coil domain containing 166	ND	ND

chr8:145063847-145064067	GRINA	glutamate receptor, ionotropic, N-methyl D-aspartate-associated protein 1 (glutamate binding)	1.08	1.10
chr9:1049663-1050336	DMRT2	doublesex and mab-3 related transcription factor 2	ND	ND
chr9:4741522-4741834	AK3	adenylate kinase 3	-1.83	2.24
chr9:85678015-85678554	RASEF	RAS and EF-hand domain containing	ND	11.52
chr9:91604847-91606106	C9orf47	chromosome 9 open reading frame 47	ND	ND
chr9:94712802-94713170	ROR2	receptor tyrosine kinase-like orphan receptor 2	ND	ND
chr9:95820393-95820841	SUSD3	sushi domain containing 3	ND	ND
chr9:112810170-112810850	AKAP2	A kinase (PRKA) anchor protein 2	ND	ND
chr9:114937639-114938268	SUSD1	sushi domain containing 1	ND	2.47
chr9:116861080-116861657	KIF12	kinesin family member 12	ND	ND
chr9:124061457-124061981	GSN	gelsolin	-1.01	1.90
chr9:124132758-124133463	STOM	stomatin	ND	ND
chr9:129566487-129567110	ZBTB43	zinc finger and BTB domain containing 43	-2.62	1.68
chr9:130158698-130159310	SLC2A8	solute carrier family 2 (facilitated glucose transporter), member 8	-3.20	3.39
chr9:130679487-130679848	ST6GALNAC4	ST6 (alpha-N-acetyl-neuraminyl-2,3-beta-galactosyl-1,3)-N-acetylgalactosaminide alpha-2,6-sialyltransferase 4	ND	ND
chr9:132082661-132082947	C9orf106	chromosome 9 open reading frame 106	ND	ND
chr9:132387648-132388295	NTMT1	N-terminal Xaa-Pro-Lys N-methyltransferase 1	-12.85	2.48
chr9:132805891-132806568	FNBP1	formin binding protein 1	-2.35	-1.50
chr9:132933990-132934306	NCS1	neuronal calcium sensor 1	-1.17	3.42
chr9:136039461-136040047	GBGT1	globoside alpha-1,3-N-acetylgalactosaminyltransferase 1	ND	ND

chrX:20135284-20136154	MAP7D2	MAP7 domain containing 2	ND	ND
chrX:30265023-30265627	MAGEB1	melanoma antigen family B, 1	ND	ND
chrX:49090257-49090734	CACNA1F	calcium channel, voltage-dependent, L type, alpha 1F subunit	ND	ND
chrX:118827547-118828401	SEPT6	septin 6	-7.16	-1.10
chrX:129245235-129245582	ELF4	E74-like factor 4 (ets domain transcription factor)	-5.71	1.53
chrX:129304888-129305345	RAB33A	RAB33A, member RAS oncogene family	ND	ND
chrX:133680285-133680770	MIR503	microRNA 503	-∞	1.33

Table S4.

Functional enrichment analysis result for 554 co-expression genes (Related to Figure S4)

GOID	Term	p-value
**Biological Process**		
GO:0090361	regulation of platelet-derived growth factor production	1.82E-18
GO:0048662	negative regulation of smooth muscle cell proliferation	9.43E-15
GO:0070373	negative regulation of ERK1 and ERK2 cascade	3.37E-12
GO:0003322	pancreatic A cell development	1.55E-11
GO:0021902	commitment of neuronal cell to specific neuron type in forebrain	1.55E-11
GO:0021905	forebrain-midbrain boundary formation	1.55E-11
GO:0021917	somatic motor neuron fate commitment	1.55E-11
GO:0021918	regulation of transcription from RNA polymerase II promoter involved in somatic motor neuron fate commitment	1.55E-11
GO:0060586	multicellular organismal iron ion homeostasis	2.43E-11
GO:0006438	valyl-tRNA aminoacylation	3.38E-11
GO:0009950	dorsal/ventral axis specification	1.05E-10
GO:0048660	regulation of smooth muscle cell proliferation	3.56E-10
GO:0021796	cerebral cortex regionalization	4.53E-10
GO:0009887	organ morphogenesis	5.52E-10
GO:0010574	regulation of vascular endothelial growth factor production	5.79E-10
GO:0009798	axis specification	8.53E-10
GO:0048568	embryonic organ development	9.09E-10
GO:0009653	anatomical structure morphogenesis	9.46E-10
GO:0021912	regulation of transcription from RNA polymerase II promoter involved in spinal cord motor neuron fate specification	1.17E-09
GO:0034587	piRNA metabolic process	2.53E-09
GO:0003310	pancreatic A cell differentiation	3.07E-09
GO:0032808	lacrimal gland development	3.07E-09
GO:0021779	oligodendrocyte cell fate commitment	4.83E-09
GO:0048562	embryonic organ morphogenesis	6.57E-09
GO:0021798	forebrain dorsal/ventral pattern formation	6.74E-09
GO:0021913	regulation of transcription from RNA polymerase II promoter involved in ventral spinal cord interneuron specification	6.74E-09
GO:0021523	somatic motor neuron differentiation	6.74E-09

GO:0021514	ventral spinal cord interneuron differentiation	1.07E-08
GO:0008285	negative regulation of cell proliferation	1.20E-08
GO:0021797	forebrain anterior/posterior pattern specification	1.51E-08
GO:0023019	signal transduction involved in regulation of gene expression	1.97E-08
GO:0042127	regulation of cell proliferation	2.54E-08
GO:0021778	oligodendrocyte cell fate specification	6.15E-08
GO:0021780	glial cell fate specification	6.15E-08
GO:0010038	response to metal ion	6.27E-08
GO:0007205	protein kinase C-activating G-protein coupled receptor signaling pathway	6.27E-08
GO:0048646	anatomical structure formation involved in morphogenesis	6.56E-08
GO:0015684	ferrous iron transport	8.66E-08
GO:0070627	ferrous iron import	8.66E-08
GO:0097286	iron ion import	8.66E-08
GO:0070372	regulation of ERK1 and ERK2 cascade	8.66E-08
GO:0034755	iron ion transmembrane transport	8.94E-08
GO:0021978	telencephalon regionalization	9.23E-08
GO:0021520	spinal cord motor neuron cell fate specification	9.76E-08
GO:0021903	rostrocaudal neural tube patterning	1.58E-07
GO:0021521	ventral spinal cord interneuron specification	4.72E-07
GO:0060573	cell fate specification involved in pattern specification	4.72E-07
GO:0006422	aspartyl-tRNA aminoacylation	5.15E-07
GO:0021983	pituitary gland development	6.01E-07
GO:0050680	negative regulation of epithelial cell proliferation	6.88E-07
GO:0021877	forebrain neuron fate commitment	6.88E-07
GO:0060579	ventral spinal cord interneuron fate commitment	6.88E-07
GO:0060581	cell fate commitment involved in pattern specification	6.88E-07
GO:0048505	regulation of timing of cell differentiation	6.88E-07
GO:0030432	peristalsis	6.88E-07
GO:0060429	epithelium development	7.15E-07
GO:0040034	regulation of development, heterochronic	1.05E-06
GO:0043009	chordate embryonic development	1.07E-06
GO:0046686	response to cadmium ion	1.07E-06
GO:0009888	tissue development	1.33E-06
GO:0002369	T cell cytokine production	1.56E-06
GO:0048708	astrocyte differentiation	1.63E-06
GO:0009790	embryo development	1.63E-06
GO:0009792	embryo development ending in birth or egg hatching	1.71E-06
GO:0048598	embryonic morphogenesis	1.73E-06
GO:0021781	glial cell fate commitment	1.73E-06
GO:0042991	transcription factor import into nucleus	1.75E-06

GO:0051531	NFAT protein import into nucleus	1.75E-06
GO:0010039	response to iron ion	1.97E-06
GO:0032502	developmental process	2.42E-06
GO:0048665	neuron fate specification	2.54E-06
GO:0060850	regulation of transcription involved in cell fate commitment	2.85E-06
GO:0021536	diencephalon development	3.35E-06
GO:0007435	salivary gland morphogenesis	3.88E-06
GO:0002052	positive regulation of neuroblast proliferation	3.94E-06
GO:0008284	positive regulation of cell proliferation	3.98E-06
GO:0071294	cellular response to zinc ion	4.04E-06
GO:0002367	cytokine production involved in immune response	4.04E-06
GO:0008016	regulation of heart contraction	4.18E-06
GO:0021513	spinal cord dorsal/ventral patterning	5.33E-06
GO:0048729	tissue morphogenesis	5.72E-06
GO:0007431	salivary gland development	7.28E-06
GO:0031099	regeneration	7.89E-06
GO:0042246	tissue regeneration	8.49E-06
GO:0014821	phasic smooth muscle contraction	8.61E-06
GO:0050678	regulation of epithelial cell proliferation	9.19E-06
GO:0048709	oligodendrocyte differentiation	9.48E-06
GO:2000179	positive regulation of neural precursor cell proliferation	1.01E-05
GO:0048732	gland development	1.02E-05
GO:0002009	morphogenesis of an epithelium	1.04E-05
GO:0061138	morphogenesis of a branching epithelium	1.05E-05
GO:0003002	regionalization	1.10E-05
GO:0035270	endocrine system development	1.17E-05
GO:0021511	spinal cord patterning	1.22E-05
GO:0021517	ventral spinal cord development	1.39E-05
GO:0009953	dorsal/ventral pattern formation	1.47E-05
GO:0001771	immunological synapse formation	1.49E-05
GO:0001763	morphogenesis of a branching structure	1.50E-05
GO:0051384	response to glucocorticoid stimulus	1.56E-05
GO:0002088	lens development in camera-type eye	1.58E-05
GO:0010042	response to manganese ion	1.61E-05
GO:0007584	response to nutrient	1.65E-05
GO:0009952	anterior/posterior pattern specification	1.75E-05
GO:0007275	multicellular organismal development	2.15E-05
GO:0048856	anatomical structure development	2.23E-05
GO:0010040	response to iron(II) ion	2.23E-05
GO:0001818	negative regulation of cytokine production	2.28E-05
GO:0030858	positive regulation of epithelial cell differentiation	2.54E-05
GO:0048754	branching morphogenesis of a tube	2.55E-05

GO:0021871	forebrain regionalization	2.55E-05
GO:0060558	regulation of calcidiol 1-monooxygenase activity	2.55E-05
GO:0060022	hard palate development	2.55E-05
GO:0031960	response to corticosteroid stimulus	2.74E-05
GO:0001709	cell fate determination	2.85E-05
GO:0045822	negative regulation of heart contraction	2.86E-05
GO:0048522	positive regulation of cellular process	3.20E-05
GO:0021532	neural tube patterning	3.21E-05
GO:0009725	response to hormone stimulus	3.39E-05
GO:0022612	gland morphogenesis	4.07E-05
GO:0035272	exocrine system development	4.13E-05
GO:0001501	skeletal system development	4.68E-05
GO:0048731	system development	4.81E-05
GO:0043409	negative regulation of MAPK cascade	4.95E-05
GO:0007389	pattern specification process	5.66E-05
GO:0007126	meiosis	6.31E-05
GO:0051327	M phase of meiotic cell cycle	6.31E-05
GO:0033993	response to lipid	6.46E-05
GO:0010035	response to inorganic substance	6.60E-05
GO:0021879	forebrain neuron differentiation	6.62E-05
GO:0031579	membrane raft organization	6.62E-05
GO:0048667	cell morphogenesis involved in neuron differentiation	6.76E-05
GO:0048705	skeletal system morphogenesis	7.66E-05
GO:0048545	response to steroid hormone stimulus	9.05E-05
GO:0051241	negative regulation of multicellular organismal process	9.29E-05
GO:0021522	spinal cord motor neuron differentiation	9.31E-05
GO:0051321	meiotic cell cycle	9.32E-05
GO:0060058	positive regulation of apoptotic process involved in mammary gland involution	9.32E-05
GO:0001878	response to yeast	9.32E-05
GO:0021515	cell differentiation in spinal cord	0.000106102
GO:0044707	single-multicellular organism process	0.000110337
GO:0048663	neuron fate commitment	0.000115851
GO:0007399	nervous system development	0.000116177
GO:0031076	embryonic camera-type eye development	0.000119035
GO:0042822	pyridoxal phosphate metabolic process	0.000123016
GO:0042823	pyridoxal phosphate biosynthetic process	0.000123016
GO:0042462	eye photoreceptor cell development	0.000124106
GO:0001754	eye photoreceptor cell differentiation	0.000124286
GO:0000904	cell morphogenesis involved in differentiation	0.000134346
GO:0030030	cell projection organization	0.00013973
GO:0032501	multicellular organismal process	0.000151139
GO:2000177	regulation of neural precursor cell proliferation	0.000152414

GO:0048706	embryonic skeletal system development	0.000162997
GO:0010741	negative regulation of intracellular protein kinase cascade	0.000162997
GO:0030856	regulation of epithelial cell differentiation	0.000167913
GO:0060675	ureteric bud morphogenesis	0.000170038
GO:0060706	cell differentiation involved in embryonic placenta development	0.000197393
GO:0031016	pancreas development	0.000197827
GO:0006418	tRNA aminoacylation for protein translation	0.00022986
GO:0030182	neuron differentiation	0.000263864
GO:0031018	endocrine pancreas development	0.000269439
GO:0001996	positive regulation of heart rate by epinephrine-norepinephrine	0.000307189
GO:0045935	positive regulation of nucleobase-containing compound metabolic process	0.000309785
GO:0048513	organ development	0.00031071
GO:0002456	T cell mediated immunity	0.000321894
GO:0048812	neuron projection morphogenesis	0.000322533
GO:0046530	photoreceptor cell differentiation	0.000331871
GO:0006939	smooth muscle contraction	0.000331871
GO:0031325	positive regulation of cellular metabolic process	0.000335522
GO:0043038	amino acid activation	0.000338216
GO:0043039	tRNA aminoacylation	0.000338216
GO:0021543	pallium development	0.000352848
GO:0051173	positive regulation of nitrogen compound metabolic process	0.0003748
GO:0048596	embryonic camera-type eye morphogenesis	0.0003748
GO:0071276	cellular response to cadmium ion	0.0003748
GO:0031328	positive regulation of cellular biosynthetic process	0.000377724
GO:0042461	photoreceptor cell development	0.000377724
GO:0010557	positive regulation of macromolecule biosynthetic process	0.000402557
GO:0000041	transition metal ion transport	0.000402557
GO:0010459	negative regulation of heart rate	0.000402557
GO:0001892	embryonic placenta development	0.000428594
GO:0048704	embryonic skeletal system morphogenesis	0.000428594
GO:0043408	regulation of MAPK cascade	0.000447185
GO:0035239	tube morphogenesis	0.000475165
GO:0051270	regulation of cellular component movement	0.000479187
GO:0006783	heme biosynthetic process	0.000482534
GO:0002762	negative regulation of myeloid leukocyte differentiation	0.000498969
GO:0006779	porphyrin-containing compound biosynthetic process	0.000544926
GO:0009891	positive regulation of biosynthetic process	0.000569266

GO:0009750	response to fructose stimulus	0.000591999
GO:0002027	regulation of heart rate	0.000595072
GO:0007409	axonogenesis	0.000611088
GO:0045823	positive regulation of heart contraction	0.000723041
GO:0010043	response to zinc ion	0.000756552
GO:0021872	forebrain generation of neurons	0.000762838
GO:0030334	regulation of cell migration	0.000815997
GO:0060562	epithelial tube morphogenesis	0.000820864
GO:0010604	positive regulation of macromolecule metabolic process	0.00083109
GO:0031175	neuron projection development	0.000840024
GO:0048048	embryonic eye morphogenesis	0.000840024
GO:0060745	mammary gland branching involved in pregnancy	0.000840024
GO:0017182	peptidyl-diphthamide metabolic process	0.000840024
GO:0017183	peptidyl-diphthamide biosynthetic process from peptidyl-histidine	0.000840024
GO:0060707	trophoblast giant cell differentiation	0.000840024
GO:0044699	single-organism process	0.000845191
GO:0048518	positive regulation of biological process	0.000845191
GO:0055072	iron ion homeostasis	0.000845191
GO:0009893	positive regulation of metabolic process	0.00092075
GO:0048666	neuron development	0.001039752
GO:0006452	translational frameshifting	0.001054645
GO:0045905	positive regulation of translational termination	0.001054645
GO:0043010	camera-type eye development	0.001090387
GO:0048871	multicellular organismal homeostasis	0.001097
GO:0032879	regulation of localization	0.001118373
GO:0042168	heme metabolic process	0.001156492
GO:0001708	cell fate specification	0.00116766
GO:0018202	peptidyl-histidine modification	0.00116766
GO:0046847	filopodium assembly	0.001231144
GO:0001658	branching involved in ureteric bud morphogenesis	0.001240926
GO:0032570	response to progesterone stimulus	0.001331494
GO:0045598	regulation of fat cell differentiation	0.001346228
GO:0032012	regulation of ARF protein signal transduction	0.001485812
GO:0031667	response to nutrient levels	0.001543994
GO:0030838	positive regulation of actin filament polymerization	0.001567712
GO:0060251	regulation of glial cell proliferation	0.001587615
GO:0042982	amyloid precursor protein metabolic process	0.001587894
GO:2000145	regulation of cell motility	0.001714494
GO:0003321	positive regulation of blood pressure by epinephrine-norepinephrine	0.001714494
GO:0060252	positive regulation of glial cell proliferation	0.001714494
GO:0045901	positive regulation of translational elongation	0.001714494

GO:0033574	response to testosterone stimulus	0.001963107
GO:0045597	positive regulation of cell differentiation	0.002129738
GO:0006778	porphyrin-containing compound metabolic process	0.00215388
GO:0051341	regulation of oxidoreductase activity	0.002338108
GO:0010288	response to lead ion	0.002355196
GO:0007127	meiosis I	0.002535885
GO:0001993	regulation of systemic arterial blood pressure by norepinephrine-epinephrine	0.002580206
GO:0006642	triglyceride mobilization	0.002580206
GO:0008612	peptidyl-lysine modification to hypusine	0.002580206
GO:0046516	hypusine metabolic process	0.002580206
GO:0009968	negative regulation of signal transduction	0.002858757
GO:0032768	regulation of monooxygenase activity	0.00287132
GO:0008217	regulation of blood pressure	0.002886351
GO:0035295	tube development	0.003028676
GO:0040012	regulation of locomotion	0.003028676
GO:0030154	cell differentiation	0.003109606
GO:0009991	response to extracellular stimulus	0.003197662
GO:0001655	urogenital system development	0.003298538
GO:0051149	positive regulation of muscle cell differentiation	0.0033498
GO:0048858	cell projection morphogenesis	0.003366506
GO:0044702	single organism reproductive process	0.003387452
GO:0003012	muscle system process	0.003387452
GO:0045663	positive regulation of myoblast differentiation	0.003390599
GO:0048268	clathrin coat assembly	0.003390599
GO:0048468	cell development	0.00343928
GO:0048519	negative regulation of biological process	0.003544615
GO:0006936	muscle contraction	0.003555136
GO:0051591	response to cAMP	0.003566024
GO:0022008	neurogenesis	0.003576929
GO:0045665	negative regulation of neuron differentiation	0.003679576
GO:0060052	neurofilament cytoskeleton organization	0.003691392
GO:0051094	positive regulation of developmental process	0.003910355
GO:0070838	divalent metal ion transport	0.003910355
GO:0045893	positive regulation of transcription, DNA-dependent	0.00401845
GO:0072179	nephric duct formation	0.00401845
GO:0046633	alpha-beta T cell proliferation	0.00401845
GO:0009719	response to endogenous stimulus	0.00402949
GO:0042493	response to drug	0.004152124
GO:0006825	copper ion transport	0.004178668
GO:0006338	chromatin remodeling	0.004253368
GO:0021915	neural tube development	0.004335561
GO:0048699	generation of neurons	0.004337116

GO:0032990	cell part morphogenesis	0.004669808
GO:0072511	divalent inorganic cation transport	0.004678439
GO:0006081	cellular aldehyde metabolic process	0.004678439
GO:0045944	positive regulation of transcription from RNA polymerase II promoter	0.004724991
GO:0001657	ureteric bud development	0.004812966
GO:0021510	spinal cord development	0.004918949
GO:0042063	gliogenesis	0.004920654
GO:0071772	response to BMP stimulus	0.004988879
GO:0071773	cellular response to BMP stimulus	0.004988879
GO:0006449	regulation of translational termination	0.004988879
GO:0048869	cellular developmental process	0.005161725
GO:0030100	regulation of endocytosis	0.005161725
GO:0051272	positive regulation of cellular component movement	0.005208003
GO:0048523	negative regulation of cellular process	0.005316775
GO:0010628	positive regulation of gene expression	0.005316775
GO:0001654	eye development	0.005316775
GO:0023057	negative regulation of signaling	0.005411102
GO:0030216	keratinocyte differentiation	0.00550124
GO:0030001	metal ion transport	0.005632098
GO:0051254	positive regulation of RNA metabolic process	0.00580427
GO:0034103	regulation of tissue remodeling	0.00580427
GO:0033014	tetrapyrrole biosynthetic process	0.00580427
GO:0070542	response to fatty acid	0.00580427
GO:0060021	palate development	0.00584314
GO:0034105	positive regulation of tissue remodeling	0.0059814
GO:0010648	negative regulation of cell communication	0.005991387
GO:0070664	negative regulation of leukocyte proliferation	0.006009854
GO:0045596	negative regulation of cell differentiation	0.006026895
GO:0003006	developmental process involved in reproduction	0.006335497
GO:0009062	fatty acid catabolic process	0.006491878
GO:0031943	regulation of glucocorticoid metabolic process	0.006516041
GO:0031334	positive regulation of protein complex assembly	0.006522291
GO:0003062	regulation of heart rate by chemical signal	0.006755728
GO:0051014	actin filament severing	0.006755728
GO:0043415	positive regulation of skeletal muscle tissue regeneration	0.006755728
GO:0043416	regulation of skeletal muscle tissue regeneration	0.006755728
GO:0046683	response to organophosphorus	0.007089496
GO:0016043	cellular component organization	0.007190755
GO:0007141	male meiosis I	0.007190755
GO:0010001	glial cell differentiation	0.007644883
GO:0034660	ncRNA metabolic process	0.007644883
GO:0060343	trabecula formation	0.007824489

GO:0090009	primitive streak formation	0.008492064
GO:0000902	cell morphogenesis	0.008642403
GO:0022407	regulation of cell-cell adhesion	0.008642403
GO:0031529	ruffle organization	0.008661491
GO:0032273	positive regulation of protein polymerization	0.008832359
GO:0048821	erythrocyte development	0.008968786
GO:0045595	regulation of cell differentiation	0.009070884
GO:0032526	response to retinoic acid	0.009293307
GO:0032989	cellular component morphogenesis	0.009317815
GO:0021987	cerebral cortex development	0.009851603
GO:0009913	epidermal cell differentiation	0.009851603
GO:0042221	response to chemical stimulus	0.009912219
GO:0032370	positive regulation of lipid transport	0.010229153
GO:0071548	response to dexamethasone stimulus	0.010242781
GO:0031944	negative regulation of glucocorticoid metabolic process	0.010417713
GO:0031947	negative regulation of glucocorticoid biosynthetic process	0.010417713
GO:0090032	negative regulation of steroid hormone biosynthetic process	0.010417713
GO:0001890	placenta development	0.010417713
GO:0032305	positive regulation of icosanoid secretion	0.010417713
GO:0060907	positive regulation of macrophage cytokine production	0.010417713
GO:0045110	intermediate filament bundle assembly	0.010417713
GO:0045654	positive regulation of megakaryocyte differentiation	0.010417713
GO:0021761	limbic system development	0.010608896
GO:0071840	cellular component organization or biogenesis	0.010697537
GO:0071396	cellular response to lipid	0.011057352
GO:0048745	smooth muscle tissue development	0.011085113
GO:0030900	forebrain development	0.011409301
GO:0071398	cellular response to fatty acid	0.011583357
GO:0050769	positive regulation of neurogenesis	0.012481916
GO:0045638	negative regulation of myeloid cell differentiation	0.012481916
GO:0051239	regulation of multicellular organismal process	0.012528336
GO:0007224	smoothened signaling pathway	0.012528336
GO:0060571	morphogenesis of an epithelial fold	0.012562622
GO:0001953	negative regulation of cell-matrix adhesion	0.01361554
GO:0048009	insulin-like growth factor receptor signaling pathway	0.01361554
GO:0072009	nephron epithelium development	0.013664099
GO:0048536	spleen development	0.01420347
GO:0009988	cell-cell recognition	0.01420347
GO:0048585	negative regulation of response to stimulus	0.014693861
GO:0065007	biological regulation	0.015032142
GO:0010940	positive regulation of necrotic cell death	0.015221337
GO:0051901	positive regulation of mitochondrial depolarization	0.015221337

GO:0007140	male meiosis	0.015352327
GO:0032945	negative regulation of mononuclear cell proliferation	0.015416414
GO:0050672	negative regulation of lymphocyte proliferation	0.015416414
GO:0050892	intestinal absorption	0.015701761
GO:0009620	response to fungus	0.015843223
GO:0032303	regulation of icosanoid secretion	0.016024583
GO:0061081	positive regulation of myeloid leukocyte cytokine production involved in immune response	0.016024583
GO:0060284	regulation of cell development	0.016989659
GO:0072329	monocarboxylic acid catabolic process	0.017223291
GO:0033013	tetrapyrrole metabolic process	0.017819049
GO:0010627	regulation of intracellular protein kinase cascade	0.01800007
GO:0030218	erythrocyte differentiation	0.01837936
GO:1901566	organonitrogen compound biosynthetic process	0.018870601
GO:0008015	blood circulation	0.018997523
GO:0050789	regulation of biological process	0.019129257
GO:0003013	circulatory system process	0.019619807
GO:0090066	regulation of anatomical structure size	0.019735389
GO:0034644	cellular response to UV	0.019735389
GO:0001701	in utero embryonic development	0.019877303
GO:0006826	iron ion transport	0.020152464
GO:0021537	telencephalon development	0.020407982
GO:0048593	camera-type eye morphogenesis	0.020445808
GO:0071248	cellular response to metal ion	0.020498523
GO:0002761	regulation of myeloid leukocyte differentiation	0.020498523
GO:0032353	negative regulation of hormone biosynthetic process	0.020848732
GO:0045989	positive regulation of striated muscle contraction	0.020848732
GO:0072178	nephric duct morphogenesis	0.020848732
GO:0060253	negative regulation of glial cell proliferation	0.020848732
GO:0032271	regulation of protein polymerization	0.021101882
GO:0051093	negative regulation of developmental process	0.022465601
GO:0007423	sensory organ development	0.022528427
GO:0072073	kidney epithelium development	0.022663088
GO:0048872	homeostasis of number of cells	0.022663088
GO:0010935	regulation of macrophage cytokine production	0.023039773
GO:0031065	positive regulation of histone deacetylation	0.023039773
GO:0001816	cytokine production	0.023042394
GO:0045671	negative regulation of osteoclast differentiation	0.023065091
GO:0030335	positive regulation of cell migration	0.023205344
GO:0006305	DNA alkylation	0.023205344
GO:0006306	DNA methylation	0.023205344
GO:0006461	protein complex assembly	0.023463552
GO:0007131	reciprocal meiotic recombination	0.023470985

GO:0035825	reciprocal DNA recombination	0.023470985
GO:0001666	response to hypoxia	0.023470985
GO:0014070	response to organic cyclic compound	0.023823086
GO:0050793	regulation of developmental process	0.024100354
GO:0070271	protein complex biogenesis	0.025088848
GO:0050794	regulation of cellular process	0.025257579
GO:0043933	macromolecular complex subunit organization	0.025257579
GO:0016331	morphogenesis of embryonic epithelium	0.025257579
GO:0010720	positive regulation of cell development	0.025257579
GO:0045055	regulated secretory pathway	0.025346588
GO:0036293	response to decreased oxygen levels	0.026431741
GO:0043046	DNA methylation involved in gamete generation	0.026541118
GO:0031115	negative regulation of microtubule polymerization	0.026694746
GO:0032351	negative regulation of hormone metabolic process	0.026694746
GO:0043171	peptide catabolic process	0.026694746
GO:0061303	cornea development in camera-type eye	0.026694746
GO:2000147	positive regulation of cell motility	0.02704238
GO:0034101	erythrocyte homeostasis	0.027385246
GO:0030521	androgen receptor signaling pathway	0.027849821
GO:0006325	chromatin organization	0.02889801
GO:0060627	regulation of vesicle-mediated transport	0.030720246
GO:0045776	negative regulation of blood pressure	0.030759885
GO:0000003	reproduction	0.030905587
GO:0030198	extracellular matrix organization	0.031061821
GO:0016042	lipid catabolic process	0.031061821
GO:2000193	positive regulation of fatty acid transport	0.031061821
GO:0046689	response to mercury ion	0.031061821
GO:0000266	mitochondrial fission	0.031061821
GO:0031063	regulation of histone deacetylation	0.031061821
GO:0002448	mast cell mediated immunity	0.031061821
GO:0045727	positive regulation of translation	0.031110155
GO:0051276	chromosome organization	0.031358291
GO:0032350	regulation of hormone metabolic process	0.03186543
GO:0045661	regulation of myoblast differentiation	0.03186543
GO:0043062	extracellular structure organization	0.031867393
GO:0009605	response to external stimulus	0.033959786
GO:0007411	axon guidance	0.034316274
GO:0031946	regulation of glucocorticoid biosynthetic process	0.034316274
GO:0007004	telomere maintenance via telomerase	0.034316274
GO:0007568	aging	0.034510158
GO:0060349	bone morphogenesis	0.035216203
GO:0032147	activation of protein kinase activity	0.035582536
GO:0072001	renal system development	0.035735439

GO:0040017	positive regulation of locomotion	0.035802025
GO:0010460	positive regulation of heart rate	0.035802025
GO:0006448	regulation of translational elongation	0.035802025
GO:0022600	digestive system process	0.036021957
GO:0022603	regulation of anatomical structure morphogenesis	0.036257489
GO:0014074	response to purine-containing compound	0.037877797
GO:0022414	reproductive process	0.038150539
GO:0060537	muscle tissue development	0.038150539
GO:0043299	leukocyte degranulation	0.039603437
GO:0007420	brain development	0.039667977
GO:0048546	digestive tract morphogenesis	0.039852297
GO:0030183	B cell differentiation	0.039852297
GO:0065003	macromolecular complex assembly	0.040196243
GO:0048608	reproductive structure development	0.040234548
GO:0061458	reproductive system development	0.040234548
GO:0044242	cellular lipid catabolic process	0.040981832
GO:0006278	RNA-dependent DNA replication	0.041168514
GO:0051101	regulation of DNA binding	0.042259801
GO:0007417	central nervous system development	0.04231304
GO:0072176	nephric duct development	0.0423332
GO:0014012	peripheral nervous system axon regeneration	0.0423332
GO:0032308	positive regulation of prostaglandin secretion	0.0423332
GO:0010939	regulation of necrotic cell death	0.0423332
GO:0043268	positive regulation of potassium ion transport	0.0423332
GO:0030855	epithelial cell differentiation	0.042404264
GO:0032270	positive regulation of cellular protein metabolic process	0.042600881
GO:0072088	nephron epithelium morphogenesis	0.042600881
GO:0051153	regulation of striated muscle cell differentiation	0.042600881
GO:0003206	cardiac chamber morphogenesis	0.043288173
GO:0008037	cell recognition	0.044110386
GO:2000026	regulation of multicellular organismal development	0.044133348
GO:0016568	chromatin modification	0.044218746
GO:0071482	cellular response to light stimulus	0.044218746
GO:0061383	trabecula morphogenesis	0.044218746
GO:0002260	lymphocyte homeostasis	0.045044934
GO:0046148	pigment biosynthetic process	0.045044934
GO:0051250	negative regulation of lymphocyte activation	0.0450513
GO:0048589	developmental growth	0.045385589
GO:0070482	response to oxygen levels	0.04545817
GO:0002444	myeloid leukocyte mediated immunity	0.045757579
GO:0022408	negative regulation of cell-cell adhesion	0.045757579
GO:0048483	autonomic nervous system development	0.047733828
GO:0009966	regulation of signal transduction	0.049283431

GO:0071822	protein complex subunit organization	0.049320665
GO:0070302	regulation of stress-activated protein kinase signaling cascade	0.049782148
Cellular Component		
GO:0033391	chromatoid body	1.13E-06
GO:0014069	postsynaptic density	1.34E-06
GO:0044327	dendritic spine head	1.34E-06
GO:0043219	lateral loop	2.55E-05
GO:0005845	mRNA cap binding complex	6.62E-05
GO:0034518	RNA cap binding complex	6.62E-05
GO:0043197	dendritic spine	7.41E-05
GO:0044309	neuron spine	7.41E-05
GO:0044456	synapse part	0.000110337
GO:0005942	phosphatidylinositol 3-kinase complex	0.000197393
GO:0005844	polysome	0.000274689
GO:0030425	dendrite	0.000402557
GO:0033267	axon part	0.000492242
GO:0031012	extracellular matrix	0.000653093
GO:0045202	synapse	0.001240926
GO:0030424	axon	0.001494181
GO:0008021	synaptic vesicle	0.001537939
GO:0045254	pyruvate dehydrogenase complex	0.001587615
GO:0035770	ribonucleoprotein granule	0.001941853
GO:0000790	nuclear chromatin	0.001949516
GO:0000785	chromatin	0.002409895
GO:0005883	neurofilament	0.002580206
GO:0005578	proteinaceous extracellular matrix	0.002886351
GO:0033268	node of Ranvier	0.003368059
GO:0060053	neurofilament cytoskeleton	0.004988879
GO:0043005	neuron projection	0.005316775
GO:0044427	chromosomal part	0.00580427
GO:0044454	nuclear chromosome part	0.006119406
GO:0071546	pi-body	0.006755728
GO:0035748	myelin sheath abaxonal region	0.006805122
GO:0000228	nuclear chromosome	0.008629013
GO:0001772	immunological synapse	0.008968786
GO:0043186	P granule	0.010417713
GO:0045495	pole plasm	0.010417713
GO:0060293	germ plasm	0.010417713
GO:0030870	Mre11 complex	0.010417713
GO:0030136	clathrin-coated vesicle	0.018870601
GO:0005581	collagen	0.021101882
GO:0031594	neuromuscular junction	0.021293067

GO:0030315	T-tubule	0.021502533
GO:0005694	chromosome	0.02189217
GO:0044420	extracellular matrix part	0.030720246
GO:0005903	brush border	0.031358291
GO:0032993	protein-DNA complex	0.036021957
GO:0005720	nuclear heterochromatin	0.0423332
GO:0005887	integral to plasma membrane	0.042600881
GO:0009986	cell surface	0.042600881
GO:0044421	extracellular region part	0.042804478
GO:0000786	nucleosome	0.045385589
GO:0042383	sarcolemma	0.04545817
Molecular Function		
GO:0008241	peptidyl-dipeptidase activity	3.24E-12
GO:0004999	vasoactive intestinal polypeptide receptor activity	1.61E-11
GO:0004832	valine-tRNA ligase activity	7.08E-11
GO:0004143	diacylglycerol kinase activity	5.45E-10
GO:0046870	cadmium ion binding	5.79E-10
GO:0071837	HMG box domain binding	1.60E-09
GO:0005381	iron ion transmembrane transporter activity	2.53E-09
GO:0008434	vitamin D3 receptor activity	4.83E-09
GO:0034584	piRNA binding	6.02E-09
GO:0002161	aminoacyl-tRNA editing activity	2.59E-08
GO:0015086	cadmium ion transmembrane transporter activity	8.66E-08
GO:0015087	cobalt ion transmembrane transporter activity	8.66E-08
GO:0015094	lead ion transmembrane transporter activity	8.66E-08
GO:0015099	nickel cation transmembrane transporter activity	8.66E-08
GO:0043565	sequence-specific DNA binding	3.14E-07
GO:0008237	metallopeptidase activity	9.16E-07
GO:0004815	aspartate-tRNA ligase activity	1.56E-06
GO:0000979	RNA polymerase II core promoter sequence-specific DNA binding	1.86E-06
GO:0035035	histone acetyltransferase binding	2.85E-06
GO:0070412	R-SMAD binding	2.85E-06
GO:0070410	co-SMAD binding	3.94E-06
GO:0004030	aldehyde dehydrogenase [NAD(P)+] activity	3.94E-06
GO:0016627	oxidoreductase activity, acting on the CH-CH group of donors	4.61E-06
GO:0003700	sequence-specific DNA binding transcription factor activity	6.22E-06
GO:0001071	nucleic acid binding transcription factor activity	6.93E-06
GO:0050897	cobalt ion binding	8.02E-06
GO:0000976	transcription regulatory region sequence-specific DNA binding	1.05E-05

GO:0005384	manganese ion transmembrane transporter activity	1.49E-05
GO:0016151	nickel cation binding	1.49E-05
GO:0001046	core promoter sequence-specific DNA binding	2.19E-05
GO:0015093	ferrous iron transmembrane transporter activity	2.55E-05
GO:0004028	3-chloroallyl aldehyde dehydrogenase activity	4.21E-05
GO:0001047	core promoter binding	4.68E-05
GO:0046915	transition metal ion transmembrane transporter activity	4.76E-05
GO:0019834	phospholipase A2 inhibitor activity	4.81E-05
GO:0035014	phosphatidylinositol 3-kinase regulator activity	4.81E-05
GO:0000981	sequence-specific DNA binding RNA polymerase II transcription factor activity	8.26E-05
GO:0046625	sphingolipid binding	9.91E-05
GO:0004812	aminoacyl-tRNA ligase activity	0.000120139
GO:0016875	ligase activity, forming carbon-oxygen bonds	0.000126345
GO:0016876	ligase activity, forming aminoacyl-tRNA and related compounds	0.000126345
GO:0005375	copper ion transmembrane transporter activity	0.00014229
GO:0005507	copper ion binding	0.00014229
GO:0043208	glycosphingolipid binding	0.000162533
GO:0046935	1-phosphatidylinositol-3-kinase regulator activity	0.000162533
GO:0000977	RNA polymerase II regulatory region sequence-specific DNA binding	0.000162997
GO:0008238	exopeptidase activity	0.000185277
GO:0005545	1-phosphatidylinositol binding	0.000185277
GO:0042393	histone binding	0.000197827
GO:0008518	reduced folate carrier activity	0.000197827
GO:0001012	RNA polymerase II regulatory region DNA binding	0.000244471
GO:0004740	pyruvate dehydrogenase (acetyl-transferring) kinase activity	0.000268599
GO:0004716	receptor signaling protein tyrosine kinase activity	0.000268599
GO:0005086	ARF guanyl-nucleotide exchange factor activity	0.000274689
GO:0005006	epidermal growth factor-activated receptor activity	0.000307189
GO:0019842	vitamin binding	0.0003748
GO:0015379	potassium:chloride symporter activity	0.000402557
GO:0016628	oxidoreductase activity, acting on the CH-CH group of donors, NAD or NADP as acceptor	0.000460275
GO:0051219	phosphoprotein binding	0.000462533
GO:0005499	vitamin D binding	0.000591999
GO:0042975	peroxisome proliferator activated receptor binding	0.000591999
GO:0016903	oxidoreductase activity, acting on the aldehyde or oxo group of donors	0.00060972
GO:0016620	oxidoreductase activity, acting on the aldehyde or oxo group of donors, NAD or NADP as acceptor	0.000780918

GO:0050291	sphingosine N-acyltransferase activity	0.000819112
GO:0004222	metalloendopeptidase activity	0.000870996
GO:0005542	folic acid binding	0.001039752
GO:0051117	ATPase binding	0.001231144
GO:0019904	protein domain specific binding	0.001729128
GO:0035257	nuclear hormone receptor binding	0.001742716
GO:0031625	ubiquitin protein ligase binding	0.001925192
GO:0044389	small conjugating protein ligase binding	0.001925192
GO:0044212	transcription regulatory region DNA binding	0.001963107
GO:0035258	steroid hormone receptor binding	0.002120826
GO:0005328	neurotransmitter:sodium symporter activity	0.00215388
GO:0003995	acyl-CoA dehydrogenase activity	0.002554748
GO:0008093	cytoskeletal adaptor activity	0.00287132
GO:0030145	manganese ion binding	0.002961324
GO:0051427	hormone receptor binding	0.003712597
GO:0001614	purinergic nucleotide receptor activity	0.003910355
GO:0016502	nucleotide receptor activity	0.003910355
GO:0003883	CTP synthase activity	0.00401845
GO:0015377	cation:chloride symporter activity	0.004178668
GO:0005326	neurotransmitter transporter activity	0.004253368
GO:0015294	solute:cation symporter activity	0.004728007
GO:0003727	single-stranded RNA binding	0.004866773
GO:0052689	carboxylic ester hydrolase activity	0.004880494
GO:0051184	cofactor transporter activity	0.005095541
GO:0003677	DNA binding	0.005214496
GO:0030331	estrogen receptor binding	0.005244209
GO:0008134	transcription factor binding	0.005518586
GO:0008289	lipid binding	0.005518586
GO:0000975	regulatory region DNA binding	0.005907302
GO:0001067	regulatory region nucleic acid binding	0.005907302
GO:0030276	clathrin binding	0.006026895
GO:0051185	coenzyme transporter activity	0.006755728
GO:0043559	insulin binding	0.006755728
GO:0060090	binding, bridging	0.006942083
GO:0030246	carbohydrate binding	0.007184865
GO:0016303	1-phosphatidylinositol-3-kinase activity	0.007190755
GO:0003993	acid phosphatase activity	0.007824489
GO:0035586	purinergic receptor activity	0.008149492
GO:0008233	peptidase activity	0.009293307
GO:0005249	voltage-gated potassium channel activity	0.009619329
GO:0035004	phosphatidylinositol 3-kinase activity	0.010242781
GO:0070567	cytidylyltransferase activity	0.010417713
GO:0046332	SMAD binding	0.01091016

GO:0070011	peptidase activity, acting on L-amino acid peptides	0.012411309
GO:0043560	insulin receptor substrate binding	0.01361554
GO:0015293	symporter activity	0.013708353
GO:0004465	lipoprotein lipase activity	0.015221337
GO:0008475	procollagen-lysine 5-dioxygenase activity	0.015221337
GO:0005078	MAP-kinase scaffold activity	0.015221337
GO:0008195	phosphatidate phosphatase activity	0.016024583
GO:0046983	protein dimerization activity	0.019631023
GO:0003997	acyl-CoA oxidase activity	0.019661719
GO:0004859	phospholipase inhibitor activity	0.019735389
GO:0051861	glycolipid binding	0.020626321
GO:0043237	laminin-1 binding	0.020848732
GO:0070815	peptidyl-lysine 5-dioxygenase activity	0.020848732
GO:0003847	1-alkyl-2-acetylglycerophosphocholine esterase activity	0.020848732
GO:0072341	modified amino acid binding	0.021340726
GO:0019207	kinase regulator activity	0.023039773
GO:0004879	ligand-activated sequence-specific DNA binding RNA polymerase II transcription factor activity	0.02373246
GO:0046873	metal ion transmembrane transporter activity	0.024985903
GO:0055102	lipase inhibitor activity	0.025346588
GO:0032947	protein complex scaffold	0.026488664
GO:0052742	phosphatidylinositol kinase activity	0.028643726
GO:0004668	protein-arginine deiminase activity	0.034316274
GO:0000983	RNA polymerase II core promoter sequence-specific DNA binding transcription factor activity	0.034316274
GO:0005215	transporter activity	0.03535247
GO:0003729	mRNA binding	0.035447458
GO:0015079	potassium ion transmembrane transporter activity	0.039852297
GO:0003713	transcription coactivator activity	0.040170396
GO:0005085	guanyl-nucleotide exchange factor activity	0.041766528
GO:0005251	delayed rectifier potassium channel activity	0.0423332
GO:0030674	protein binding, bridging	0.044133348
GO:0048038	quinone binding	0.0450513
